# Single-cell transcriptome analysis and protein profiling reveal broad immune system activation in IgG4-related disease

**DOI:** 10.1172/jci.insight.167602

**Published:** 2023-09-08

**Authors:** Chenyang Lu, Shasha Li, Pingying Qing, Qiuping Zhang, Xing Ji, Zhigang Tang, Chunyan Chen, Tong Wu, Yidan Hu, Yi Zhao, Xiaohui Zhang, Qi He, David A. Fox, Chunyu Tan, Yubin Luo, Yi Liu

**Affiliations:** 1 Department of Rheumatology and Immunology, West China Hospital, Sichuan University, Chengdu, China.; 2 Division of Rheumatology, Department of Internal Medicine, and; 3 Guangdong Provincial Key Laboratory of Diabetology & Guangzhou Municipal Key Laboratory of Mechanistic and Translational Obesity Research, Medical Center for Comprehensive Weight Control, The Third Affiliated Hospital of Sun Yat-sen University, Guangzhou, China.; 4 State Key Laboratory of Oral Diseases, National Clinical Research Center for Oral Diseases, West China Hospital of Stomatology, Sichuan University, Chengdu, China.; 5 Division of Rheumatology and Clinical Autoimmunity Center of Excellence, Department of Internal Medicine, University of Michigan, Ann Arbor, Michigan, USA.

**Keywords:** Autoimmunity, Inflammation, Adaptive immunity, Bioinformatics, Immunoglobulins

## Abstract

IgG4-related disease (IgG4-RD) is a systemic autoimmune disease with unclear pathogenesis. We performed single-cell RNA-seq and surface proteome analyses on 61,379 PBMCs from 9 treatment-naive IgG4-RD patients and 7 age- and sex-matched healthy controls. Integrative analyses were performed for altered gene expression in IgG4-RD, and flow cytometry and immunofluorescence were used for validation. We observed expansion of plasmablasts with enhanced protein processing and activation, which correlated with the number of involved organs in IgG4-RD. Increased proportions of CD4^+^ cytotoxic T lymphocytes (CTLs), CD8^+^ CTLs-GNLY (granulysin), and γδT cells with enhanced chemotaxis and cytotoxicity but with suppressed inhibitory receptors characterize IgG4-RD. Prominent infiltration of lymphocytes with distinct compositions were found in different organs of IgG4-RD patients. Transcription factors (TFs), including PRDM1/XBP1 and RUNX3, were upregulated in IgG4-RD, promoting the differentiation of plasmablasts and CTLs, respectively. Monocytes in IgG4-RD have stronger expression of genes related to cell adhesion and chemotaxis, which may give rise to profibrotic macrophages in lesions. The gene activation pattern in peripheral immune cells indicated activation of multiple interaction pathways between cell types, in part through chemokines or growth factors and their receptors. Specific upregulation of TFs and expansion of plasmablasts and CTLs may be involved in the pathogenesis of IgG4-RD, and each of these populations are candidate targets for therapeutic interventions in this disease.

## Introduction

IgG4-related disease (IgG4-RD) is an insidiously progressive inflammatory and fibrosing condition characterized by tumefactive lesions, dense lymphoplasmacytic infiltrates, and abundant IgG4^+^ plasma cells in the affected tissues. Affecting approximately 0.28–1.08/100,000 individuals, IgG4-RD is a newly recognized rare disease (1, 2). Common histological features can be found in nearly every organ, including the lacrimal and salivary glands, pancreas, hepatobiliary tract, kidneys, and lymph nodes (3). The disease can lead to permanent organ injury and even death if left untreated (4).

Previous studies have demonstrated that both innate and adaptive immune cells are involved in the pathogenesis of IgG4-RD (5). As a hallmark of IgG4-RD, CD19^+^CD27^+^CD20^–^CD38^hi^ plasmablasts are significantly clonally expanded, with extensive somatic mutation (6). The proportion of circulating plasmablasts correlates with the number of involved organs and disease activity and facilitates assessment of patients’ response to treatment; however, its relationship with serum levels of IgG4 is still under debate (7, 8). Tissue examination of IgG4-RD lesions revealed that CD4^+^SLAMF7^+^ cytotoxic T lymphocytes (CTLs) and granzyme A–expressing CD8^+^ CTLs were the dominant infiltrating T cells, and circulating CD4^+^ CTLs decreased with improvement of symptoms (9–11). Moreover, the predominant infiltration of M2 macrophages in multiple organs was thought to play an important role in the fibrosis noted in IgG4-RD lesions (12, 13). Other immune cells such as basophils, mast cells, plasmacytoid dendritic cells (pDCs), and some T cell subsets are also involved (5). Despite the increased knowledge, factors regulating these immune cells and the exact pathophysiology underlying this fibroinflammatory condition remain enigmatic. Advances in high-resolution single-cell RNA sequencing (scRNA-seq) have provided an avenue to identify disease-related cell subsets and explore transcriptional features at a cellular resolution in blood and tissue samples.

In this study, we apply scRNA-seq, together with antibody-oligonucleotide conjugates (AbSeq), to characterize the cell composition, gene signature, developmental trajectories, and regulators of immune cells in peripheral blood (PB) from patients with IgG4-RD.

## Results

### Single-cell multiomics analysis of peripheral immune cells.

**Figure 1:**
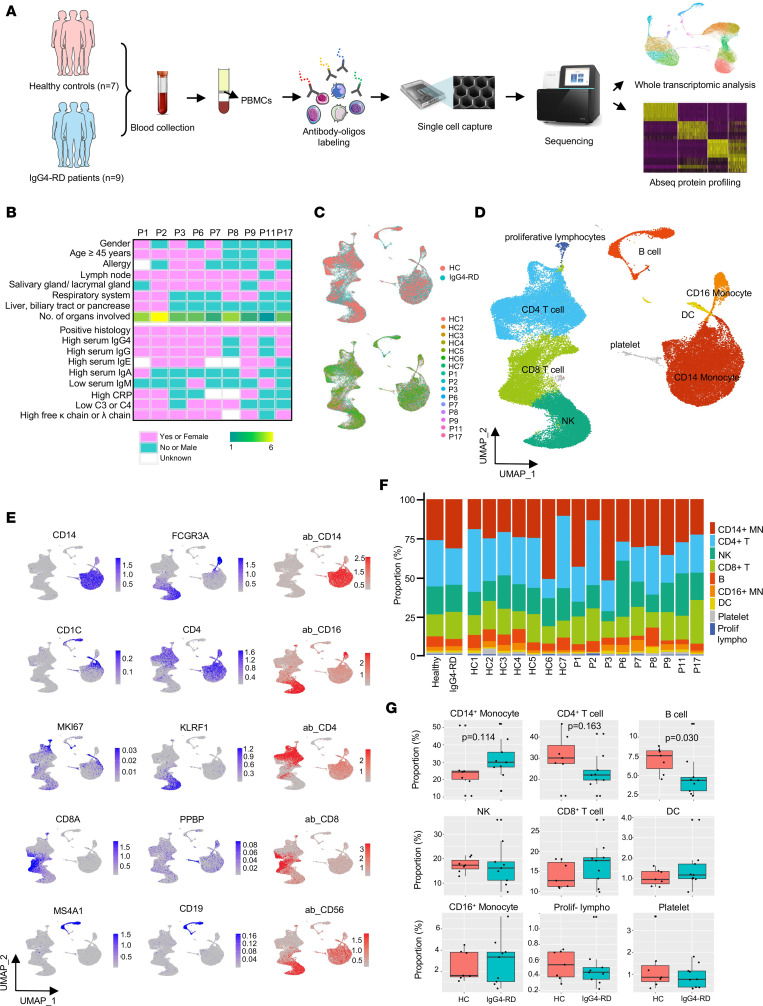
Single-cell multiomics analysis of PBMCs from individuals with IgG4-RD and healthy controls. (**A**) Overview of the participants included and the samples and data collected. (**B**) Description of vital clinical characteristics of patients. (**C**) UMAP visualization of 61,379 cells after QC according to groups and samples, respectively. (**D**) UMAP plot of clustering determined by Seurat v.4 shows a total of 9 major clusters (clusters 0 to 8) that were identified and color coded. (**E**) Projection of cells expressing transcripts (blue) and chosen surface proteins (red) to the UMAP plots. (**F**) Bar plot of the proportions of cell types shown in **D** separated by condition and donor. HC, healthy control; P, patient; MN, monocyte; NK, natural killer cell; DC, dendritic cell; Prolif lympho, proliferating lymphocytes. (**G**) Comparison of proportions of each cell type between the 2 groups (7 HCs, 9 IgG4-RD patients). Shown are exact 2-tailed *P* values by Wilcoxon’s rank-sum test (CD14^+^ monocyte, B, DC, platelet, and prolif. lympho) or Student’s *t* test (CD3^+^ T, NK, CD8^+^ T, and CD16^+^ monocyte) according to distribution.w Box-and-whisker plot features: minimum whisker, 25th percentile − 1.5 × interquartile range (IQR) or the lowest value within; minimum box, 25th percentile; center, median; maximum box, 75th percentile; maximum whisker, 75th percentile + 1.5 × IQR or greatest value within.

To characterize the immunological features of IgG4-RD, we performed scRNA-seq and AbSeq in PBMCs from 9 IgG4-RD patients and 7 age- and sex-matched healthy controls (HCs) (Figure 1, A and B, and Supplemental Tables 1 and 2; supplemental material available online with this article; https://doi.org/10.1172/jci.insight.167602DS1). After quality control and filtering, 61,379 cells, including 21,851 from HCs and 39,528 from patients, were obtained (Supplemental Figure 1A). Nine distinct subsets were identified according to the transcriptional programs and surface protein expression of canonical markers (Figure 1, C–E, and Supplemental Figure 1B). These subsets include CD14^+^ monocytes (MNs) (LYZ^+^CD14^+^), CD16^+^ MNs (LYZ^+^FCGR3A^+^), DCs (CD1C^+^), CD4^+^ T cells (CD4^+^), CD8^+^ T cells (CD8A^+^), proliferating lymphocytes (MKI67^+^), natural killer (NK) cells (KLRF1^+^), B cells (CD19^+^), and platelets (PPBP^+^) (Figure 1E). Of note, the percentages of B cells and CD4^+^ T cells tended to be lower in IgG4-RD patients than in HCs, while CD14^+^ MNs showed an opposite trend (Figure 1, F and G), which was also demonstrated in a recent study (14).

### B cells are prone to differentiate into plasmablasts in IgG4-RD.

**Figure 2:**
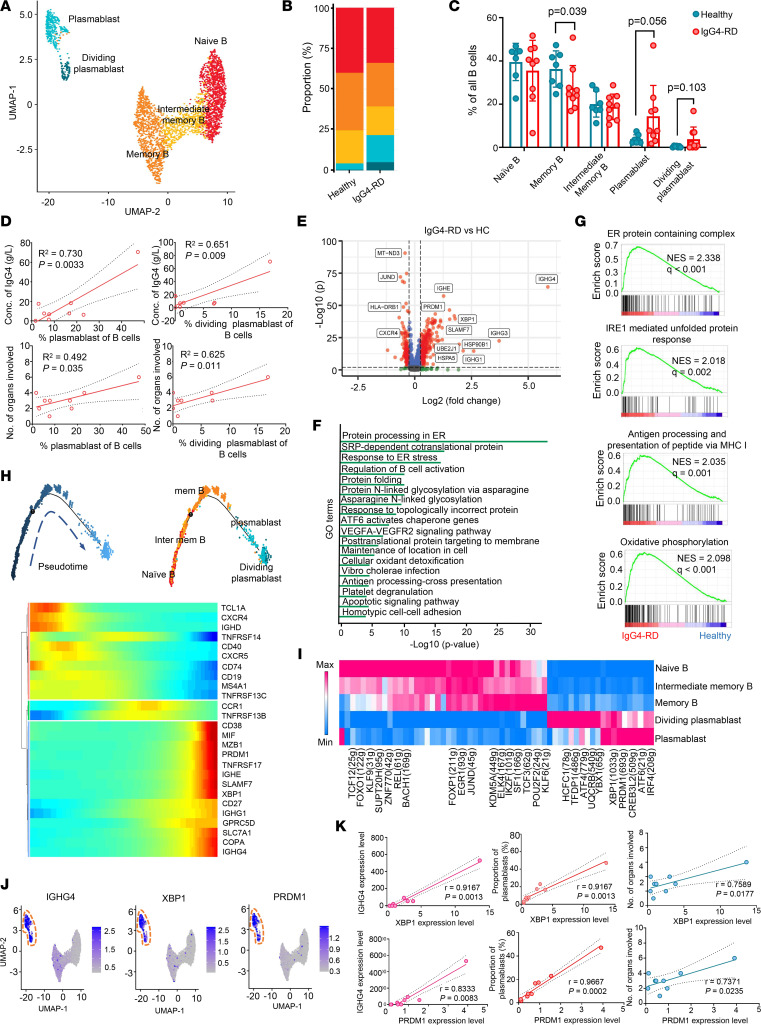
The transcriptional features of B cells in IgG4-RD. (**A**) UMAP visualization of B cell subsets shows 5 clusters that were identified and color coded. (**B** and **C**) Comparison of the proportions of B cell subtypes, separated by condition and donor. HC *n* = 7, IgG4-RD *n* = 9. HC, healthy control; P, patient. Results are shown as mean ± SD. Exact 2-sided *P* values are given. Significance was determined by Student’s *t* test (naive B, intermediate memory B) or Mann-Whitney test (memory B, plasmablast, and dividing plasmablast). (**D**) Scatter plots depicting the correlation between serum concentration of IgG4 (g/L) or numbers of organs involved and percentage of plasmablast/dividing plasmablast of B cells in IgG4-RD (*n* = 9). Shown are *R*^2^, exact 2-tailed *P* values, and 95% confidence intervals. Correlation was determined by linear regression. (**E**) Volcano plot showing the differentially expressed genes between B cells from IgG4-RD and HCs. (**F**) GO analysis using Metascape for genes that were upregulated in B cells from IgG4-RD versus HCs. (**G**) GSEA shows top enriched pathways in IgG4-RD. NES, normalized enrichment score. (**H**) Trajectory of differentiation from naive B cells to plasmablasts predicted by monocle and heatmap show upregulated or downregulated genes in the differentiation process. (**I**) Heatmap of the *t* values of AUC scores of expression regulation by transcription factors of the clusters, as estimated using SCENIC (see Supplemental Methods). (**J**) UMAP visualization of *IGHG4*, *XBP1*, and *PRDM1* in B cells. (**K**) Correlation of expression of *XBP1* and *PRDM1* with *IGHG4* expression (Spearman’s *r*), proportion of plasmablasts (Spearman’s *r*), and number of involved organs (Pearson’s *r*) in IgG4-RD (*n* = 9), respectively. Exact 2-tailed *P* values and Pearson’s/Spearman’s *r* are presented.

Given the rise of IgG4^+^ plasma cells found in IgG4-RD lesions and their critical role in the pathogenesis, a more precise and detailed understanding of the features of B cells in circulation is essential. We identified 5 transcriptionally distinct B cell subsets, including naive B (MS4A1^+^IGHD^+^TCL1A^+^), memory B (MS4A1^+^CD27^+^), intermediate memory B (IGHD^+^CD27^+^), plasmablast (MZB1^+^), and dividing plasmablasts (MZB1^+^MKI67^+^) (Figure 2A and Supplemental Figure 2, A–C). Notably, the proportion of the memory B subset decreased significantly in patients with IgG4-RD compared with HCs, while plasmablasts and dividing plasmablasts showed an increasing trend (Figure 2, B and C, and Supplemental Figure 2D), suggesting a possible transition from memory B to antibody-secreting plasmablasts in IgG4-RD. Concentrations of IgG4 were positively correlated with proportions of plasmablast and dividing plasmablasts (Figure 2D). Proportions of plasmablasts and dividing plasmablasts were positively correlated with organs involved, whereas concentrations of IgG4 or ratios of IgG4/IgG and IgG4/IgM were not (Figure 2D and Supplemental Figure 2E). This finding is consistent with the prior finding of poor diagnostic utility of serum IgG4 or IgG4/IgG in IgG4-RD (15), and indicates that the roles of plasmablasts/dividing plasmablasts in the pathogenesis of the disease are not fully reflected in systemic immunoglobin (Ig) levels.

Gene Ontology (GO) analysis showed that genes involved in protein processing in endoplasmic reticulum (ER), ER stress response, and protein folding were significantly upregulated, suggesting enhanced protein synthesis during related processes in IgG4-RD (Figure 2, E and F). This observation was validated by gene set enrichment analysis (GSEA) (Figure 2G). Interestingly, oxidative phosphorylation was highly enriched in IgG4-RD (Figure 2G), suggesting a high consumption of energy to support protein synthesis. Moreover, both GO analysis and GSEA showed enrichment of genes involved in antigen processing and presentation via MHC class I (cross presentation), indicating the role of B cells in activation of CD8^+^ T cells in IgG4-RD (Figure 2G).

Notably, Ig-encoding genes, including *IGHG4*, *IGHG1*, and *IGHE* were most highly upregulated in IgG4-RD (Figure 2E). Single-cell validation showed that *IGHG4* and *IGHE*, but not *IGHG1*, were significantly upregulated in plasmablasts/dividing plasmablasts in IgG4-RD patients compared with HCs (Supplemental Figure 2F), which is in line with elevated serum IgG4 and IgE in these patients (4). Furthermore, higher expression of ER stress–related genes such as *HSP90B1*, *HSPA5*, and *UBE2J1* were found in IgG4-RD patients (Figure 2E and Supplemental Figure 2F). B cell activation pathways were also enriched, and representative genes, including *XBP1*, *PRDM1*, and *BLK* were highly expressed in B cells from IgG4-RD (Supplemental Figure 2F). B cell survival cytokines, including B cell–activating factor (BAFF, encoded by *TNFSF13B*) and a proliferation-inducing ligand (APRIL, encoded by *TNFSF13*), are survival factors for B cells that also control B cell maturation (16). Transmembrane activator and CAML interactor (TACI/*TNFRSF13B*), BAFF receptor (BAFFR/*TNFRSF13C*), and B cell maturation antigen (BCMA/*TNFRSF17*) are closely related members of the TNF receptor superfamily and bind BAFF and APRIL (17). Interestingly, the expression levels of TACI and BAFFR were lower in IgG4-RD than in HCs, and BCMA was highly expressed in B cells, especially plasmablasts and dividing plasmablasts from IgG4-RD compared with HCs (Supplemental Figure 2G). *TNFSF13B* was highly expressed in MNs and DCs, and its expression was higher in IgG4-RD (Supplemental Figure 2H). These favor the altered homeostasis in B cell development in IgG4-RD. Consistent with our study (Supplemental Figure 2I), 2 other data sets showed that signatures of mature B cells were more enriched in blood and tissues of IgG4-RD, and were repressed with treatment (18, 19).

To understand the source of plasmablasts/dividing plasmablasts and regulatory mechanisms involved in expansion of these cells in IgG4-RD, trajectory analysis and TF analysis were applied. There was a transition from naive B, intermediate memory B, and memory B cells to IgG4-secreting plasmablasts and dividing plasmablasts (Figure 2H). During this process, markers of plasma cells, including *CD38*, *MZB1*, and Ig-coding genes were upregulated (Figure 2H). *CD74*, *CXCR4*, and *CXCR5* were decreased along stages of B cell maturation, while *MIF*, *COPA*, and *SLC7A1* were upregulated gradually, indicating that distinct chemokines were involved in chemotaxis of B cells at different stages (Figure 2H). CXCR4/CXCL12 promote the retention of immune cells in the bone marrow, while CCR2 and CX3CR1 direct their movement from circulation to the tissue (20). Interestingly, plasmablasts/dividing plasmablasts downregulated *CXCR4* but upregulated *CCR2* and *CX3CR1* (Supplemental Figure 2J), which promotes their trafficking to inflamed tissues in a coordinated, step-wise fashion.

Distinct TFs were expressed in 5 B cell subsets (Figure 2I). For example, *TFDP1*, *ATF4*, and *UQCRB* were significantly expressed in dividing plasmablasts, while *IRF4*, *CREB3L2*, and *PRDM1* were upregulated in plasmablasts (Figure 2I). TFs such as ATF4, UQCRB, and TFDP1 are involved in the regulation of cell self-renewal (21, 22). Dividing plasmablasts with high activity of these TFs could be an active source of plasma cells. IRF4 (23, 24) and PRDM1 (25) were reported to control the differentiation of plasmablasts. Moreover, CREB3L2 has been recently reported to be strongly related to the transition from a B cell to a plasma cell state (26). We found that *IGHG4*, *XBP1*, and *PRDM1* were coexpressed by plasmablasts/dividing plasmablasts (Figure 2J), and the expression levels of *XBP1* and *PRDM1* were positively correlated with *IGHG4*, proportion of plasmablasts, and marginally with the number of organs involved (Figure 2K). Therefore, XBP1 and PRDM1 may play critical roles in regulating B cell development toward plasmablasts, and could be candidate biomarkers for IgG4-RD.

### Expansion of highly chemotactic and cytotoxic T subpopulations in IgG4-RD.

**Figure 3:**
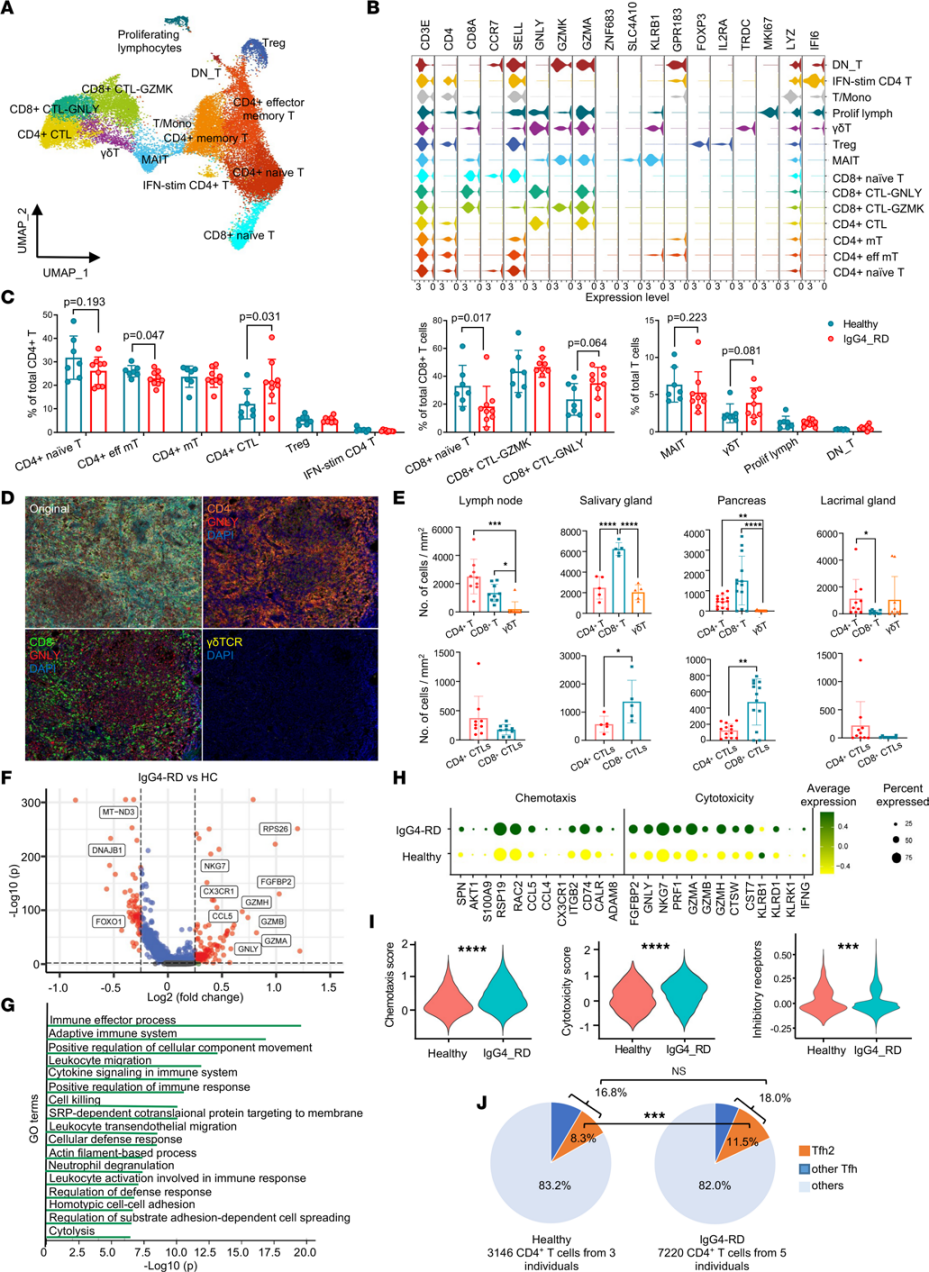
Expansion and tissue infiltration of cytotoxic T subsets in IgG4-RD. (**A**) UMAP plot of subgroups of T cells. CTL, cytotoxic T lymphocyte; Treg, regulatory T cell; MAIT, mucosal-associated invariant T cell; DN_T, double negative T cell; T/Mono, doublets of T cells and monocytes. (**B**) Violin plot showing expression distribution of canonical cell markers in cell types shown in **A**. (**C**) Comparison of proportions of each cell type between the 2 groups (7 HCs, 9 IgG4-RD patients). Results are shown as mean ± SD. Exact 2-tailed *P* values are given. Significance was determined by 2-tailed Student’s *t* test (CD4^+^ naive T, CD4^+^ effector memory T, CD4^+^ memory T, CD4^+^ CTL, Treg, IFN-stimulated CD4^+^ T, CD8^+^ CTL-GZMK, CD8^+^ CTL-GNLY, and DN_T) or Mann-Whitney test (CD8^+^ naive T, MAIT, γδT, and prolif. lymph). (**D**) Representative tissue multicolor immunofluorescence images and modeling analyses (repeated 3 times for each tissue type). Original magnification, ×200. (**E**) Comparisons of infiltrated T cell subsets in different tissues (lymph node *n* = 9, salivary gland *n* = 5, pancreas *n* = 13, lacrimal gland *n* = 10). Kruskal-Wallis test followed by post hoc Dunn’s multiple-comparison test was performed. (**F**) Volcano plot showing the differentially expressed genes between T cells from IgG4-RD and HCs. (**G**) GO analysis using Metascape for genes that were upregulated in T cells from IgG4-RD. (**H**) The expression levels of well-defined chemotaxis- and cytotoxicity-related genes. (**I**) The chemotaxis score, cytotoxicity score, and score of inhibitory receptors in T cell subpopulations. Significance determined by Mann-Whitney test. (**J**) Constituent ratios of follicular helper T (Tfh) cells and type 2 Tfh (Tfh2) cells among CD4^+^ T cells (3 HCs, 5 IgG4-RD patients). Significance determined by χ^2^ test. *P < 0.05; **P < 0.01; ***P < 0.001; ****P < 0.0001.

T cells are the dominant cell type in the lymphoplasmacytic infiltrate in IgG4-RD. To understand circulating T cells, we subdivided T cells into 14 subsets: 6 subtypes of CD4^+^ T (CD3E^+^CD4^+^), 3 subtypes of CD8^+^ T (CD3E^+^CD8A^+^), γδT (CD3E^+^CD4^–^CD8A^–^TRDC^+^), mucosal-associated invariant T (MAIT) cells (CD3E^+^SLC4A10^+^), proliferating lymphocytes (CD3E^+^MKI67^+^), double-negative T (DN_T, CD3E^+^CD4^–^CD8^–^), and T/MN doublets (Figure 3, A and B, and Supplemental Figure 3, A and B). Of the 6 subtypes of CD4^+^ T cell clusters, in addition to naive CD4^+^ T cell (CCR7^+^SELL^+^), memory CD4^+^ T cell (GPR183^+^), effector memory CD4^+^ T cell (GPR183^+^KLRB1^+^), and regulatory T (Treg) cell (IL21RA^+^FOXP3^+^) subtypes, we defined 2 activated CD4^+^ T subtypes, CD4^+^ CTL (GZMA^+^ ZNF683^+^) and IFN-stimulated CD4^+^ T (IFI6^+^). The CD4^+^ CTL cluster was characterized by high expression of genes associated with cytotoxicity, including *GNLY* and *GZMA* (Figure 3B). The 3 subtypes of CD8^+^ T cell included a naive CD8^+^ T subset (CCR7^+^ SELL^+^) and 2 effector CD8^+^ T subsets (CD8^+^ CTL-GZMK and CD8^+^ CTL-GNLY), which had high expression levels of *GZMA* and *NKG7*. In detail, CD8^+^ CTL-GZMK uniquely expressed *GZMK*, whereas CD8^+^ CTL-GNLY showed relatively high expression levels of *GZMB*, *GZMH*, and *GNLY* (Figure 3, A and B). Proportions of CD4^+^ CTLs, CD8^+^ CTL-GNLY, and γδT cells increased, while proportions of CD4^+^ naive T, CD4^+^ effector memory T cells, CD8^+^ naive T, and MAIT cells decreased in patients with IgG4-RD compared with HCs (Figure 3C and Supplemental Figure 3C). Consistent with this result, higher proportions of CD4^+^ CTLs, CD8^+^ CTL-GNLY, and γδT cells were detected in PB of IgG4-RD by flow cytometry in a larger cohort (Supplemental Figure 3, D and E). In addition, the CD4^+^ CTLs and γδT cells had upregulated granzyme A expression, while CD8^+^ CTL-GNLY did not (Supplemental Figure 3E). The expansion of cytotoxic T subsets and reduction of naive T subsets in PB of IgG4-RD indicate that T cells may be activated and differentiated into cell-killing subsets.

In tissues, distinct components of infiltrated T cells were found in different organs (Figure 3, D and E). CD4^+^ and CD8^+^ T were predominant T cells in lesional sites, whereas the inflamed tissues only had a small number of γδT cells. In addition, a higher frequency of CD8^+^ T cells than CD4^+^ T cells was found in the salivary glands and pancreas, while it was opposite in lymph nodes and lacrimal glands. The constituent ratio of CD4^+^ CTL and CD8^+^ CTL-GNLY in tissues was similar.

Genes involved in immune processes, migration, cytokine signaling, and cell killing were significantly upregulated in T cells in IgG4-RD patients compared with HCs (Figure 3, F and G). T subsets, especially CD4^+^ CTL, CD8^+^ CTL-GNLY, CD8^+^ CTL-GZMK, MAIT, γδT, and proliferating lymphocytes have high chemotaxis scores; IgG4-RD had a significantly higher chemotaxis score and enhanced expression of related genes than HCs, suggesting T cells in PB of IgG4-RD patients receive stronger migration signals (Figure 3, H and I, and Supplemental Figure 4A). Meanwhile, CD4^+^ CTL, CD8^+^ CTL-GNLY, CD8^+^ CTL-GZMK, MAIT, and γδT subsets showed high cytotoxicity scores (Figure 3, H and I, and Supplemental Figure 4A). Within these highly cytotoxic clusters, IgG4-RD had a significantly higher cytotoxicity score together with higher expression of cytotoxicity-associated genes, except *KLRB1*, compared with HCs (Figure 3H and Supplemental Figure 4B). Consistently, 2 other data sets also showed increased chemotaxis and cytotoxicity signals in both tissues (GSE40568) and blood (GSE66465) from IgG4-RD patients (Supplemental Figure 4C). In contrast, the CTLs together with γδT showed lower expression of inhibitory receptors, including TIGIT, CD244, PECAM1, and PDCD-1 (Figure 3H and Supplemental Figure 4, A and D). The protein level of PD-1 was validated with AbSeq data (Supplemental Figure 4E). We also found the cytotoxic T subsets have high expression of FGFBP2 and CX3CR1 (Supplemental Figure 4F). FGFBP2 is an important modulator of fibroblast growth factor (FGF) signaling by chaperoning FGFs through the extracellular matrix to FGF receptors (27). High prevalence of FGFBP2 variants was also found in IgG4-RD (28). Meanwhile, these cells express the fractalkine receptor CX3CR1, which has been associated with fibrosis in many organs (29). Therefore, these expanded cytotoxic T subsets are likely to migrate to tissues and promote fibrosis.

Follicular helper T cells (Tfh) are a special subset of CD4^+^ T cells that expedite B cell and plasma cell differentiation, resulting in germinal center formation. Among them, the number of type 2 Tfh (Tfh2) cells was found to be specifically increased in IgG4-RD and was correlated with serum IgG4 levels and plasmablast counts (30). Through our single-cell AbSeq data (Supplemental Figure 4G), IgG4-RD patients had a higher proportion of Tfh2 cells (11.5%) (CD45RA^–^CD4^+^PDCD1^+^CXCR5^+^CXCR3^–^CCR6^–^) than HCs did (8.3%) (*P* < 0.001), but not a significant elevation of Tfh cells (CD45RA^–^CD4^+^PDCD1^+^CXCR5^+^) (18.0%) compared with HCs (16.8%) (*P* > 0.05) (Figure 3J). However, the levels of *IL21* and *CXCL13* mRNA in these circulating Tfh cells were very low in this data set (data not shown). Treg cytokines such as IL-10 and TGF-β are proposed to be involved in the class switching of plasma cells and fibrosis in IgG4-RD. We found that the proportion of Treg cells was comparable between IgG4-RD and HCs; however, the expression of FOXP3 was higher in Treg cells in IgG4-RD (Supplemental Figure 4H), which is in line with published data (31). The RNA expression of IL-10 and TGF-β in these circulating Treg cells was barely detectable (data not shown). We speculated that these cells might expand in lymphoid organs and are finally activated locally in the lesional sites.

Moreover, GSEA showed that pathways related to the defense response to Gram-negative bacteria and regulation of B cell differentiation were upregulated in T cell genes in IgG4-RD (Supplemental Figure 4I), suggesting that T cells in IgG4-RD might be activated by some pathogens and facilitated B cell differentiation to plasmablasts. TOR signaling and Toll-like receptor signaling pathways exhibited similar trends (Supplemental Figure 4I). Although no direct evidence has demonstrated that IgG4-RD is caused by infection, gut microbiota disturbance may be a trigger for the immune dysregulation in IgG4-RD (32, 33).

**Figure 4:**
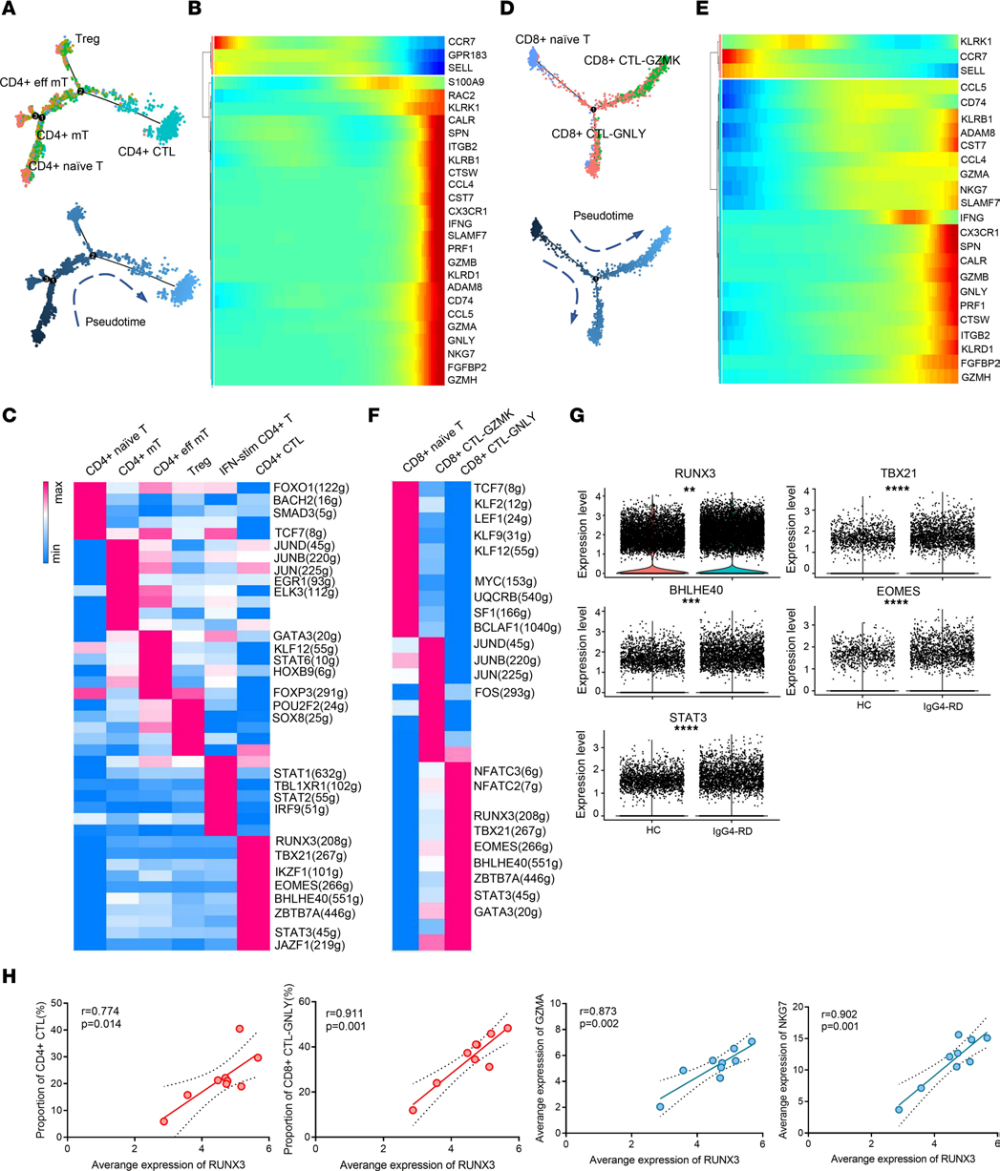
The development and vital transcription factors of T cells in IgG4-RD. Trajectory of differentiation and heatmap show upregulated or downregulated genes in the differentiation process of CD4^+^ T (**A** and **B**) and CD8^+^ T cells (**D** and **E**). Heatmap of the *t* values of AUC scores of expression regulation by transcription factors of the CD4^+^ T cells (**C**) and CD8^+^ T cells (**F**), as estimated using SCENIC (see Supplemental Methods). (**G**) Comparison of vital transcription factors between IgG4-RD and HCs. Significance was determined by Mann-Whitney test. **P < 0.01, ***P < 0.001, ****P < 0.0001. (**H**) Correlation of RUNX3 expression with proportion of CTLs and expression of GZMA and NKG7, respectively (*n* = 9). Exact 2-tailed *P* values and Pearson’s *r* are presented.

During the transition from naive T, memory T/effector memory T, all the way to CTL within CD4^+^ T cells (Figure 4A), genes involved in cytotoxicity such as *GZMA*, *GZMB*, and *GNLY*, and chemotaxis such as *CCL4*, *CCL5*, and *CX3CR1* were upregulated (Figure 4B). Among CD8^+^ T cells, naive cells developed into 2 types of effector cells and had a similar pattern in terms of chemotaxis and cytotoxicity as CD4^+^ T cells (Figure 4, D and E). CD4^+^ T subsets and CD8^+^ T subsets can be distinguished by different groups of TFs. We noticed that RUNX3, TBX21, EOMES, and BHLHE40 were specifically expressed in both CD4^+^ and CD8^+^ CTLs (Figure 4, C and F). RUNX3 is essential for T cells acquiring a cytotoxic phenotype (34), EOMES drives the expression of IFN-γ and cytotoxic molecules such as granzymes and perforins (35), and BHLHE40 is a key regulator of cytokine production by T cells, enhancing IFN-γ production while depressing IL-10 production (36). Compared with HCs, enhanced expression of RUNX3, TBX21, EOMES, BHLHE40, and STAT3 was observed in IgG4-RD, representing regulators of CTLs in IgG4-RD (Figure 4G). Additionally, there was a positive correlation of RUNX3 with proportions of CD4^+^ CTL and CD8^+^ CTL-GNLY and expression of cytotoxic granules such as GZMA and NKG7 (Figure 4H). Thus, RUNX3 and other TFs can be potential therapeutic targets for IgG4-RD.

### Features of other immune cells in PBMCs from IgG4-RD.

**Figure 5:**
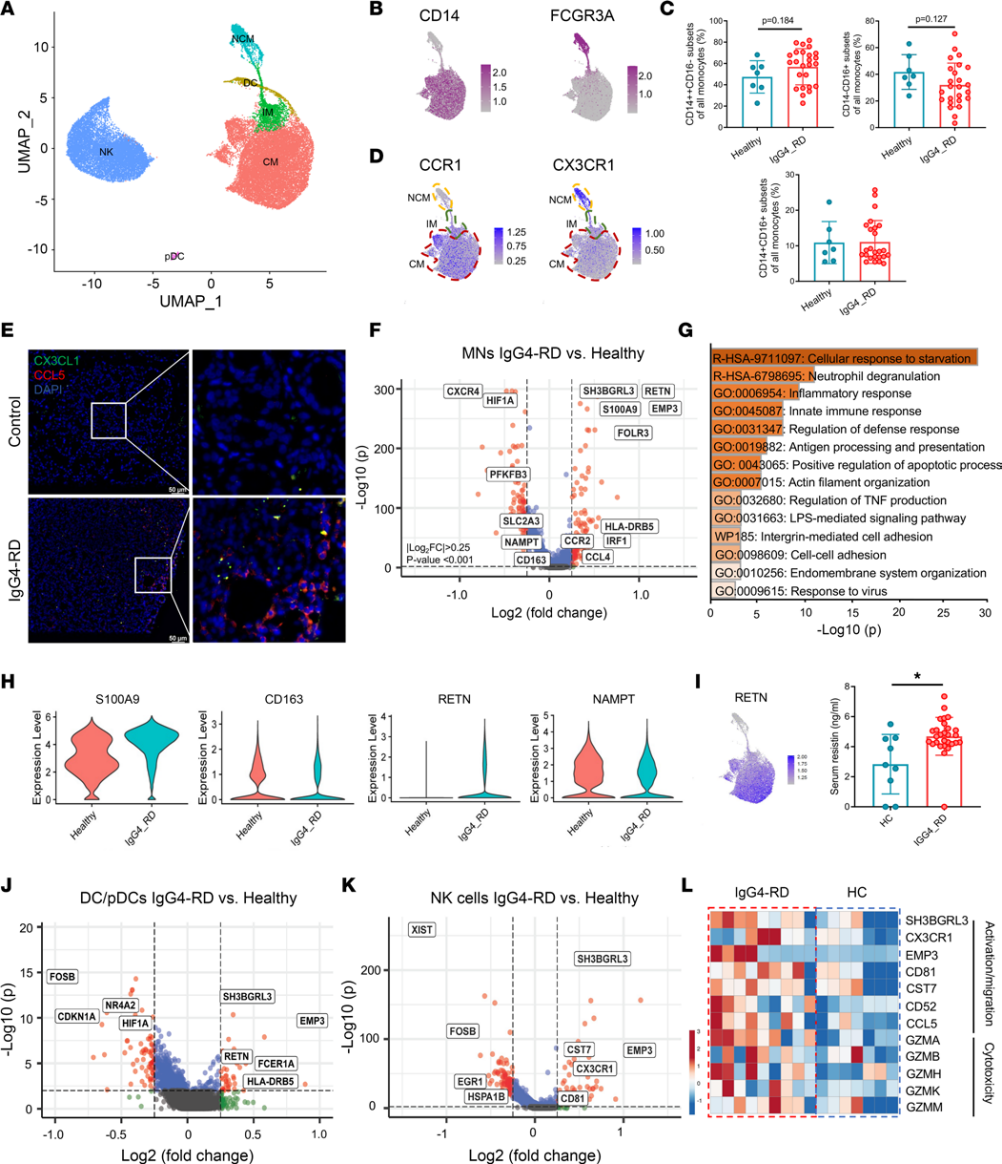
Detailed characterization of other immune cells in IgG4-RD. (**A**) UMAP plot of subgroups of other immune cells. CM, classical monocyte; IM, intermediate monocyte; NCM, nonclassical monocyte; NK, natural killer cell; DC, dendritic cell; pDC, plasmacytoid dendritic cell. (**B**) Projection of cells expressing CD14 and FCGR3A to the UMAP plots. (**C**) Flow cytometry showing proportions of CMs, IMs, and NCMs in HCs (*n* = 7) and IgG4-RD patients (*n* = 25). Significance was determined by Student’s *t* test (CD14^++^CD16^–^ MNs and CD14^–^CD16^++^ MNs) or Mann-Whitney test (CD14^+^CD16^+^ MNs). (**D**) Projection of cells expressing CCR1 and CX3CR1 to the UMAP plots. (**E**) Representative immunofluorescence for CX3CL1 and CCL5 in salivary glands from controls and IgG4-RD patients (repeated 3 times for each group). Scale bars: 50 μm. (**F**) Volcano plot showing the differentially expressed genes (DEGs) between monocytes from IgG4-RD and HCs. (**G**) GO analysis using Metascape for upregulated genes in monocytes from IgG4-RD versus HCs. (**H**) Violin plots showing expression levels of indicated genes in monocytes of IgG4-RD and HCs. (**I**) *RETN* was mainly expressed in CMs in the single-cell data and serum resistin levels were elevated in IgG4-RD patients (*n* = 28) compared with HCs (*n* = 9). Significance was determined by Mann-Whitney test. (**J**) Volcano plot showing the DEGs in DCs/pDCs from IgG4-RD compared with HCs. (**K**) Volcano plot showing the DEGs in NK cells from IgG4-RD compared with HCs. (**L**) Heatmap showing expression levels of cell activation-, migration-, and cytotoxicity-associated genes in NK cells from IgG4-RD and HCs. Results are shown as mean ± SD. Exact 2-tailed *P* values are given. Significance was determined by Student’s *t* test or Mann-Whitney test. *P < 0.05.

In addition to B and T cells, innate immune cells in PBMCs also contribute to the development of this disease. Those cells were subdivided into 6 subsets: 3 MN subsets that include classical MNs (CD14^++^FCGR3A^–^, CMs), nonclassical MNs (CD14^–^FCGR3A^++^, NCMs), intermediate MNs (CD14^+^FCGR3A^+^, IMs), 2 DC subsets — DC (CD1C^+^) and pDC (LILRA4^+^) — and NK cells (NKG7^+^) (Figure 5, A and B, and Supplemental Figure 5A). We further examined the proportions of 3 MN subsets in a larger population and found CMs had an increasing trend, while NCMs had a decreasing trend in IgG4-RD patients compared with HCs (Figure 5C and Supplemental Figure 5B). To explore whether MNs have a strong migration potential, we checked all sequenced chemokine receptors and found that both CMs and IMs had higher CCR1 expression, while NCMs had relatively higher CX3CR1 expression (Figure 5D and Supplemental Figure 5C). However, no significant difference was found in the CCR1 and CX3CR1 expression in 3 MN subsets between IgG4-RD and HCs, except that CX3CR1 expression was lower in IgG4-RD CMs compared with HC CMs (Supplemental Figure 5D). In contrast, the levels of CX3CL1 (ligand for CX3CR1) and CCL5 (a ligand for CCR1) were upregulated in IgG4-RD tissues, indicating elevated chemokines in tissues could be important factors for the migration of different MN subsets (Figure 5E).

In general, genes involved in immune response, antigen processing and presentation, and cell migration/adhesion pathways such as *RETN*, *SH3BGRL3*, *S100A9*, *EMP3*, and *CCL4* were significantly upregulated, while *CD163* and some glycolysis-related genes were downregulated in IgG4-RD MNs compared with HCs (Figure 5, F and G). Higher proportion of S100A9^+^CD163^–^ cells among CD14^+^CD16^–^ MNs was found to be associated with lung fibrosis (37). In this study, higher S100A9 but lower CD163 expression was found in IgG4-RD MNs and stronger signals of MNs and profibrotic macrophages (M2) in IgG4-RD tissues compared with HCs (Figure 5F and Supplemental Figure 5E). This suggests that there may exist a phenotype switch in MNs/macrophages at different stages. Moreover, resistin (encoded by *RETN*) is a proinflammatory, profibrotic cytokine that induces fibroblast-myofibroblast differentiation (38), while visfatin (encoded by *NAMPT*) has opposite effects (39). Compared with MNs from HCs, *RETN* was upregulated in MNs (predominantly expressed in CMs) from IgG4-RD, and serum resistin was elevated in IgG4-RD patients (Figure 5, H and I). In contrast, *NAMPT* was downregulated in MNs from IgG4-RD (Figure 5H). The imbalance of *RETN* and *NAMPT* in MNs may play a part in regulating the activation of fibroblasts and prompting the inflammatory and profibrotic environment when MNs migrated to IgG4-RD lesions.

Similar to MNs, upregulated genes in DCs and pDCs from IgG4-RD patients were enriched in cell chemotaxis, immune response, and inflammation (Figure 5J and Supplemental Figure 5F). In detail, chemotaxis-associated genes, including *CCR2*, *CX3CR1*, *FCER1G*, and *TPM4* were upregulated in IgG4-RD DCs (Supplemental Figure 5G). For NK cells, upregulated differentially expressed genes (DEGs) in IgG4-RD were enriched in immune response and cell killing pathways (Figure 5K and Supplemental Figure 5H). NK cell activation–related genes such as *CD81*, *CD52*, and *CST7* and cytotoxicity-associated genes such as *GZMA*, *GZMB*, and *GZMH* were upregulated in IgG4-RD patients compared with HCs (Figure 5L).

### Cell-cell interactions among immune cells of IgG4-RD.

**Figure 6:**
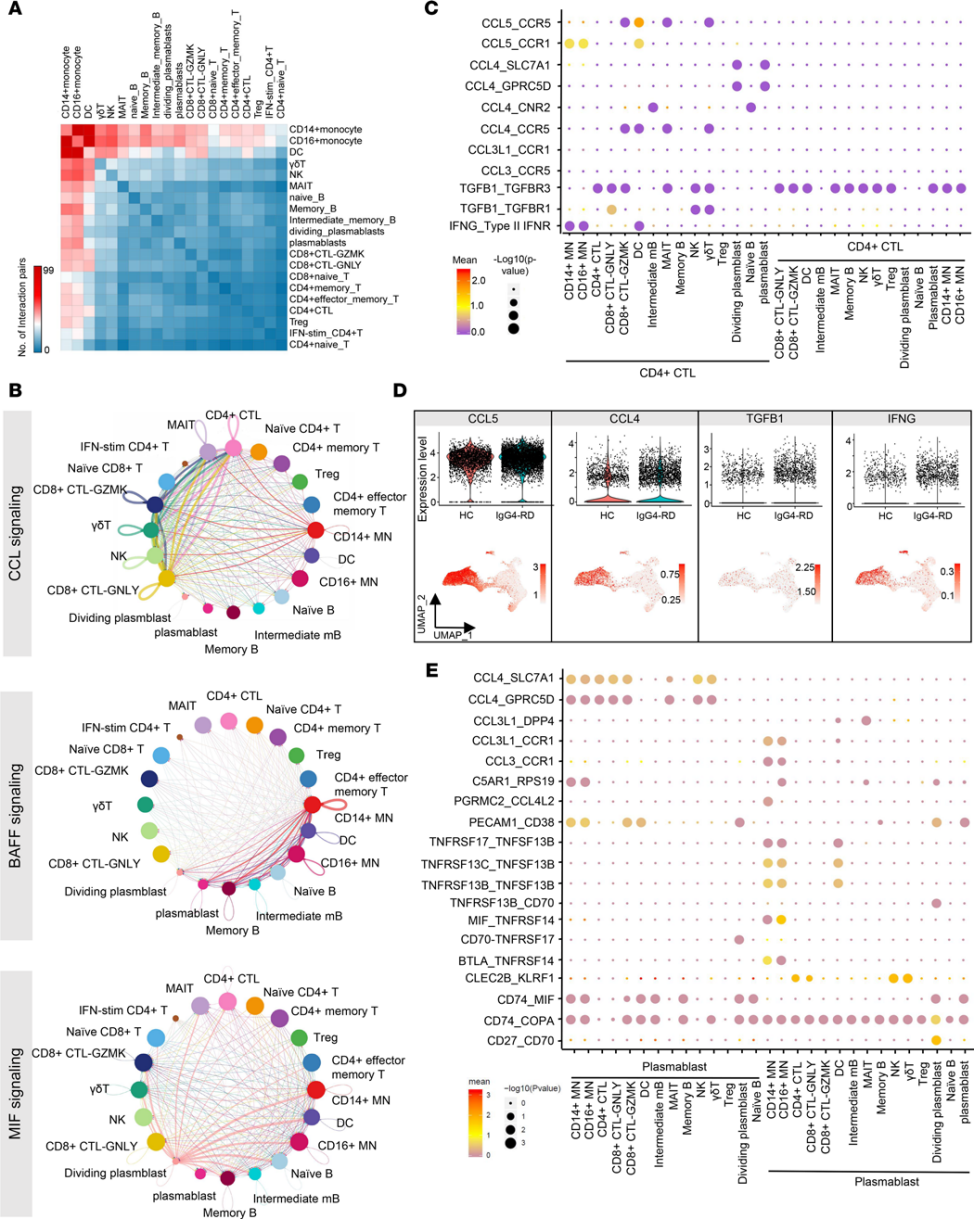
Cell-cell communication network among PBMCs. (**A**) Heatmap show number of potential ligand-receptor pairs between cell groups predicted by CellphoneDB. (**B**) Communication networks among PBMCs in terms of CCL signaling, BAFF signaling, and MIF signaling. (**C**) Bubble plots show ligand-receptor pairs between CD4^+^ CTLs and other cell types, and comparison of important cytokines in CD4^+^ CTL between IgG4-RD and HCs (**D**). (**E**) Ligand-receptor pairs of activation and chemotaxis between plasmablasts and other cell types.

We investigated the interaction network among cell types identified in PBMCs using CellphoneDB (40) and CellChat (41). CD14^+^ MNs, CD16^+^ MNs, and DCs showed the most interactions with other cells, especially among each other (Figure 6A). Cell-cell interaction based on chemokine (C-C motif) ligands (CCLs) occurred predominantly among CD14^+^ MNs, CD4^+^ CTLs, CD8^+^ CTL-GZMK, CD8^+^ CTL-GNLY, γδT, MAIT, and NK cells (Figure 6B). BAFF-based interaction was mainly found between MNs and 5 B cell subsets, while macrophage migration inhibitory factor–based (MIF-based) interaction was observed between dividing plasmablasts and others (Figure 6B). Furthermore, we found CD4^+^ CTL interactions with CD14^+^ MNs, CD16^+^ MNs, and DCs via CCL5-CCR1/CCR5, while CD4^+^ CTLs interacted with CD8^+^ CTL-GZMK, MAIT, and γδT cells via CCL4/CCL5-CCR5. Moreover, CCL5 in T/NK cells was positively correlated with CCR1 in MNs (Supplemental Figure 5I).

Plasmablasts and dividing plasmablasts were more likely to utilize CCL4-SLC7A1/GPRC5D to interact with CD4^+^ CTLs (Figure 6C). As expected, CCL4 and CCL5 were specifically expressed in CTLs and were upregulated in IgG4-RD compared with HCs (Figure 6D). Plasmablasts could employ CCL4-SLC7A1/GPRC5D and CD74-MIF/COPA to interact with other immune cells for chemotaxis (Figure 6E). Binding to CD74 on B cells, MIF is a B cell chemokine that might be responsible for the migration of pathogenic B cells to IgG4-RD manifestation sites as well as their aberrant proliferation (42). We also found signals of MN/DC–B cell crosstalk via TNFSF13B-TNFRSF17/TNFRSF13B/TNFRSF13C, which might facilitate development of plasmablasts (Figure 6E). Altogether, the interaction data revealed that abnormal B and T subsets present in IgG4-RD display a strong interaction with each other via CD74, CCL4, and CCL5, which confer signals contributing to the abnormal inflammatory responses in IgG4-RD.

## Discussion

This study created a comprehensive overview of immunological changes in IgG4-RD. We identified markers of plasmablasts and CTLs in IgG4-RD, which were associated with their differentiation and function. Importantly, TFs and cell-cell interaction of critical cell types were analyzed, contributing to the understanding and future precision therapy of IgG4-RD.

In IgG4-RD, B cells are of clear pathogenic importance, as evidenced by the efficacy of B cell depletion therapy (43) and their direct profibrotic role (44). Strong activation of B cells was observed in IgG4-RD, evidenced by expansion of plasmablasts/dividing plasmablasts. There were also positive correlations of proportions of dividing plasmablasts and plasmablasts with serum IgG4 levels and number of organs involved. Ig production in the ER together with oxidative phosphorylation, which supplies energy, are enhanced in B cells. In addition to protein processing, antigen presentation was also enhanced in B cells from IgG4-RD, which potentially contributes to the activation of T cells. The decrease in CD4^+^ CTLs in response to depletion of B cells confirmed the potential interaction between B cells and effector T cells (9). Signals favoring plasmablast survival such as TNFSF13B-TNFRSF17 were enhanced in IgG4-RD. The high expression levels of chemokine receptors CX3CR1 and CCR2 but low CXCR4 expression allow plasmablasts/dividing plasmablasts to migrate to inflamed tissues and create a vicious circle. Moreover, we found enhanced differentiation of plasmablasts and dividing plasmablasts together with higher expression of their key TFs, including XBP1 and PRDM1. The expression levels of these genes were positively correlated with extent of disease involvement. Therefore, the TFs regulating the maturation of autoreactive B cells could act as alternative therapeutic targets in this disease.

Previous studies have revealed that cytotoxic T subtypes, especially CD4^+^ CTLs, play an important role in the pathogenesis of IgG4-RD. These cells are clonally expanded both in the PB and at the lesional sites. The number of tissue-infiltrating CD4^+^ CTLs correlates with the extent of organ involvement (9, 11, 45). In the inflammatory environment, CTLs may induce apoptosis of mesenchymal cells and secrete profibrotic factors, amplifying tissue fibrosis and organ dysfunction (10). In addition to CD4^+^ CTLs, we also noticed expansion and activation of other cytotoxic effector T subsets, including CD8^+^ CTL-GNLY and γδT. These CTLs have enhanced cytotoxicity and chemotaxis, suggesting that they may have synergistic effects in inducing apoptosis of mesenchymal cells and in promoting fibrosis. We also detected different distributions of T cell subsets in various tissues; however, the actual reason for the tissue preference remains unclear. It could be due to a combination of distinct chemokine profiles in tissues, special tissue composition, and antigen specificity. The T cell repertoire had been explored in both peripheral CD4^+^ T and submandibular gland T cells from IgG4-RD (46, 47). Due to the small sample size and high disease heterogeneity, no specific pattern was observed in the V gene and J gene usage, and overlapping TCR clones were rarely found among different patients (46, 47). The features in the TCR repertoire of IgG4-RD may need further study. Through trajectory analyses, CTLs are terminally differentiated T cells. They were exquisitely regulated by a series of TFs, including RUNX3, TBX21, and EOMES. Among them, RUNX3 expression was closely related to the proportions of CTLs as well as cytotoxic factors. However, mechanisms underlying the activation of these TFs and initiation of CTL development in IgG4-RD warrant future study.

IgG4-RD patients have a slight increase in the number of CD14^+^ MNs. As the main source of BAFF in PB, CD14^+^ MNs help preserving the survival of B cells. While migrating to inflamed tissues, these cells might develop into profibrotic macrophages, as evidenced by enrichment of M2 macrophage markers. In addition to their contribution to fibrosis through production of profibrotic factors, including CCL18 and IL-10, M2 macrophages initiate Th2 immune responses via IL-33 secretion (12, 13). Inhibition of MN migration or blockage of M2 polarization may influence tissue damage.

Our study has 2 main limitations. Firstly, only PBMCs were sequenced in this study. Polymorphonuclear cells, including neutrophils and eosinophils, also play an important role in IgG4-RD (5). Also, sequencing of lesional tissues at a single-cell level could provide more details of immune responses directly. Secondly, small sample size and detection sensitivity may limit the generalization of our findings. Some genes were undetected or barely detected at the RNA level. Further studies with a larger sample size and high-throughput screening strategy are warranted.

In conclusion, we demonstrate that peripheral immune cells were broadly activated in IgG4-RD. Increased numbers of MNs were found in the circulation, which showed stronger chemotaxis signals. B cells are activated and have a higher transformation rate to Ig-secreting plasmablast/dividing plasmablast in IgG4-RD. TFs such as XBP1 and PRDM1 may be responsible for this transformation. Cytotoxic T subpopulations, including CD4^+^ CTLs, CD8^+^CTL-GNLY, and γδT were expanded in IgG4-RD. These cells have stronger cell-killing ability, and chemotaxis, and could be more profibrotic. Broad communication exists within the peripheral immune system. Understanding these pathways will not only guide clinicians in the diagnosis of rare autoimmune disorders but also facilitate the development of targeted treatments.

## Methods

### Patients and clinical characteristics.

Nine patients with IgG4-RD who fulfill 2019 ACR/EULAR classification diagnostic criteria together with 7 age- and sex-matched HCs without any known infection, autoimmune diseases, cancer, or cardiovascular diseases were recruited. The clinical data and disease course of the 9 patients are summarized in Supplemental Tables 1 and 2 and Figure 1B.

### Single-cell sequencing and analyses.

PB samples were obtained from participants and PBMCs were isolated by gradient centrifugation with Ficoll-Paque (GE Healthcare) within 4 hours (cell viability >93%). All granulocytes were depleted. PBMCs were labeled with 9 antibodies conjugated with polyadenylated and antibody-specific barcodes (Supplemental Table 3). The BD Rhapsody system was used following the manufacturer’s instructions. Sequencing libraries were generated using a nano-well–based system following the AbSeq protocol reported previously (48). The output filtered gene expression matrices were analyzed by R software (v.4.0.4) with the Seurat package (v.4.0, https://satijalab.org/seurat) according to a standard protocol (Supplemental Methods). RNA expression microarray data of labial salivary glands (GSE40568) (18) and blood (GSE66465) (19) from IgG4-RD patients were used to explore gene expression patterns in tissues or blood.

### Flow cytometry.

PBMCs were stimulated with PMA (50 ng/mL, Sigma-Aldrich) and ionomycin (1 μg/mL, Sigma-Aldrich) in the presence of protein transport inhibitor cocktail (Thermo Fisher Scientific) for 6 hours. Cells were collected and stained with BV510-conjugated anti-CD3 (catalog 740202), APC-Cy7–conjugated anti-CD4 (catalog 557871), PerCP-Cy5.5–conjugated anti-CD8 (catalog 560662), Alexa Fluor 647–conjugated anti-CD319 (catalog 564338), and BV421-conjugated γδTCR antibody (catalog 744870) for surface markers. Cells were fixed and permeabilized before staining with Alexa Fluor 488–conjugated anti-GNLY (catalog 558254) and PE-conjugated anti-GZMA (catalog 558904). After 3 washes, cells were measured using a BD flow cytometer. For MNs, cells were measured after staining with APC-Cy7–anti-CD14 (catalog 557831), BV510–anti-CD16 (catalog 563830), BV421–anti-HLA-DR (catalog 562805), APC–anti-CCR1 (catalog 362908, BioLegend), and PE–anti-CX3CR1 (catalog 355704, BioLegend). All antibodies except anti-CCR1 and anti-CX3CR1 were purchased from BD Biosciences.

### Immunofluorescence.

Formalin-fixed lymph nodes, salivary glands, pancreas, and lacrimal glands biopsies from IgG4-RD patients were paraffin-embedded. Slides were deparaffinized in xylene and antigens were retrieved. Then, the sections were stained with anti-CD4 (catalog 93518, Cell Signaling Technology), anti-CD8 (catalog 85336, Cell Signaling Technology), anti-GNLY (catalog ER61826, Huabio), and anti-γδTCR (catalog TCR1061, Thermo Fisher Scientific) according to the manufacturer’s protocol (Opal Polaris, Akoya Bioscience) and then were screened and analyzed with the Vectra Polaris Automated Quantitative Pathology Imaging system (PerkinElmer). For CCL5 and CX3CL1 staining, antibodies against CCL5 (catalog ET1705-70, Huabio) and CX3CL1 (catalog 60339-1-Ig, Proteintech) were used as primary antibodies, while Alexa Fluor 647– and 488–conjugated anti-IgG (catalog ab150079 and ab150113, Abcam) were used as secondary antibodies. Salivary gland biopsies from patients with mucocele as controls. The images were captured with a Leica microscope.

### ELISA.

Cytokines in the sera were measured using ELISA kits from CUSABIO (CSB-E06884h) according to the manufacturer’s instructions.

### Statistics.

Cell proportions are expressed as mean ± SD. Normality test was conducted to determine whether the data are normally distributed or skewed. Normally distributed data were analyzed by 2-tailed Student’s *t* test, while skewed data were analyzed by 2-tailed Mann-Whitney test or Kruskal-Wallis test. For multiple tests, Kruskal-Wallis test followed by post hoc Dunn’s multiple-comparison test was performed. Comparison of proportions of Tfh in combined data was performed by χ^2^ test. The relationship between IgG4 concentration and percentage plasmablasts/dividing plasmablasts among B cells was explored by linear regression. Pearson’s or Spearman’s correlation was performed to calculate the correlation coefficients of 2 factors according to normality of distribution. Data analyses were performed with GraphPad Prism software version 7.0. A *P* value of less than 0.05 was considered significant. DEGs between patients and HCs were analyzed with Wilcoxon’s rank-sum test implemented in Seurat. A permutation test for the CellphoneDB analysis was used to evaluate the significance of a ligand/receptor pair.

### Study approval.

This study was approved by the ethics committee of the West China Hospital of Sichuan University (no. 2020-759) and written informed consent was obtained from each participant prior participation.

### Data availability.

The data that support the findings of this study are available within the article and its Supplemental Tables and Figures. The raw scRNA-seq data have been deposited in the Genome Sequence Archive in National Genomics Data Center, China National Center for Bioinformation/Beijing Institute of Genomics, Chinese Academy of Sciences (GSA-Human: HRA003750) that are publicly accessible at https://ngdc.cncb.ac.cn/gsa-human

## Author contributions

CL, DAF, CT, and Y Luo designed the study design, interpreted data, and wrote the manuscript. CL, PQ, CT, XJ, YH, XZ, QH, and YZ collected clinical data and samples. CL, PQ, and QZ isolated PBMCs and performed flow cytometry. CL, ZT, TW, and CC stained tissue sections and performed histological examination. CL and SL conducted bioinformatic analyses. CT, Y Luo, and Y Liu supervised the whole project. All authors contributed to the manuscript and approved the submitted version. CL, SL, and PQ contributed equally. CL was listed first because he organized the data for development of the manuscript in addition to planning and leading the experiments.

## Supplementary Material

Supplemental data

Supporting data values

## Version 1 08/10/2023

In-Press Preview

## Version 2 09/08/2023

Electronic publication
