# Supplementary material for: Single-cell transcriptome analysis and protein profiling reveal broad immune system activation in IgG4-related disease
Source: JCI Insight. 2023 Sep 8;8(17):e167602. doi: 10.1172/jci.insight.167602 (PMC10544205; doi:10.1172/jci.insight.167602)
Supplement: Supplemental data [file jciinsight-8-167602-s116.pdf]

Supplementary Methods

Library building and data processing

BD Rhapsody system was used following the manufacturer's instructions. Sequencing libraries were generated using a nano-well based system following the AbSeq protocol reported previously[1]. The clustering of the index-coded samples was performed on a cBot Cluster Generation System using TruSeq PE Cluster Kit v3-cBot-HS (Illumina) according to the manufacturer's instructions. After cluster generation, the library preparations were sequenced on an Illumina Novaseq platform and 150 bp paired-end reads were generated.

Raw gene expression matrices were generated for each sample by the Cell Ranger (v.3.0.2) pipeline coupled with human reference version GRCh38. The output filtered gene expression matrices were analyzed by R software (v.4.0.4) with the Seurat package (v.4.0, https://satijalab.org/seurat). In brief, gene expressed at a proportion >0.1% of the data and cells with >200 genes detected were selected for further analyses. Low-quality cells were removed if they: 1) <800 unique molecular identifiers (UMIs); 2) <200 genes; or 3) >25% UMIs derived from the mitochondrial genome. After removal of low-quality cells, gene expression matrices were normalized by the NormalizeData function and 2000 features with high cell-to-cell variation were calculated using FindVariableFeatures function. To reduce dimensionality of the datasets, the *RunPCA* function was conducted with default parameters on linear-transformation scaled data generated by the *ScaleData* function. Next, the *ElbowPlot* functions were used to identify the true dimensionality of each dataset, as recommended by the Seurat developers. Finally, we clustered cells using the *FindNeighbors* and *FindClusters* functions and performed nonlinear dimensional reduction with the *RunUMAP* function with default settings.

After that, we identified "anchors" between individual datasets with the *FindIntegrationAnchors* function and inputted these anchors into the *IntegrateData* function to create a batch-corrected expression matrix of all cells, which allowed cells from different datasets to be integrated and analyzed together. Expression levels of 9 cell surface proteins were also generated for PBMCs from 5 IgG4-RD patients (P1, P2, P3, P4, and P17) and 3 HCs (HC5, HC6, HC7). Seurat v.4.0 was used to integrate the individual mRNA and cell surface protein expression profiles for 8 samples, generating an integrate matrix of transcriptomic and proteomic features.

Cell type annotation and cluster marker identification

After nonlinear dimensional reduction and projection of all cells into two-dimensional space by UMAP, cells were clustered together according to common features. The *FindAllMarkers* function in Seurat was used to find markers for each of the identified clusters. Clusters were then classified and annotated based on expressions of canonical markers of particular cell types. Clusters expressing two or more canonical cell type markers were classified as doublet cells and excluded from further analysis.

DEG identification and functional enrichment

Differentially expressed gene (DEG) testing was performed using the *FindMarkers* function in Seurat with parameter "test.use=wilcox" by default and the Benjamini-Hochberg method was used to estimate the false discovery rate (FDR). DEGs were filtered using a minimum $\log_2$(fold change) of 0.25 and a maximum p value of 0.001. Enrichment analysis for the functions of the DEGs was conducted using the Metascape webtool (www.metascape.org). Gene set enrichment analysis was

performed with 1000 permutations with the GSEA software (v4.1.0) on a matrix of all genes.

Defining cell state scores

The *AddModuleScore* function in Seurat was used to implement the method with default settings. We used chemotaxis-associated genes (SPN, AKT1, S100A9, RPS19, RAC2, CCL5, CCL4, CX3CR1, ITGB2, CD74, CALR, ADAM8) and cytotoxicity-associated genes (FGFBP2, GNLY, NKG7, PRF1, GZMA, GZMB, GZMH, CTSW, CST7, KLRB1, KLRD1, KLRK1, IFNG) to define the chemotaxis and cytotoxicity of T cell subpopulations. Inhibitory receptors including TIGIT, CTLA4, PDCD1, CD274, BTLA, CD160, LAG3, HAVCR2, CD244, CD200R1, and PECAM1 were used to evaluate the inhibitory signals on cytotoxic T cells.

Trajectory analysis and SCENIC analysis

Trajectory analysis was carried out using the *OrderCells* function of Monocle 2[2], setting the naïve cells, identified by canonical markers, as root node. This was done separately for B cells, CD4+ T cells, and CD8+ T cells. The SCENIC analysis was run using the pyscenic (version 0.9.19) and hg19-500bp-upstream-10species databases for RcisTarget, GRNboost, and AUCell[3]. The input normalized expression matrix was from Seurat.

Cell-cell interaction

Cell-cell interaction analysis was performed by using the CellPhoneDB package[4] and Cellchat[5]. The existence of the potential interactions between two cell types was explored by evaluating the expression levels of annotated ligands and receptors between the two cell types.

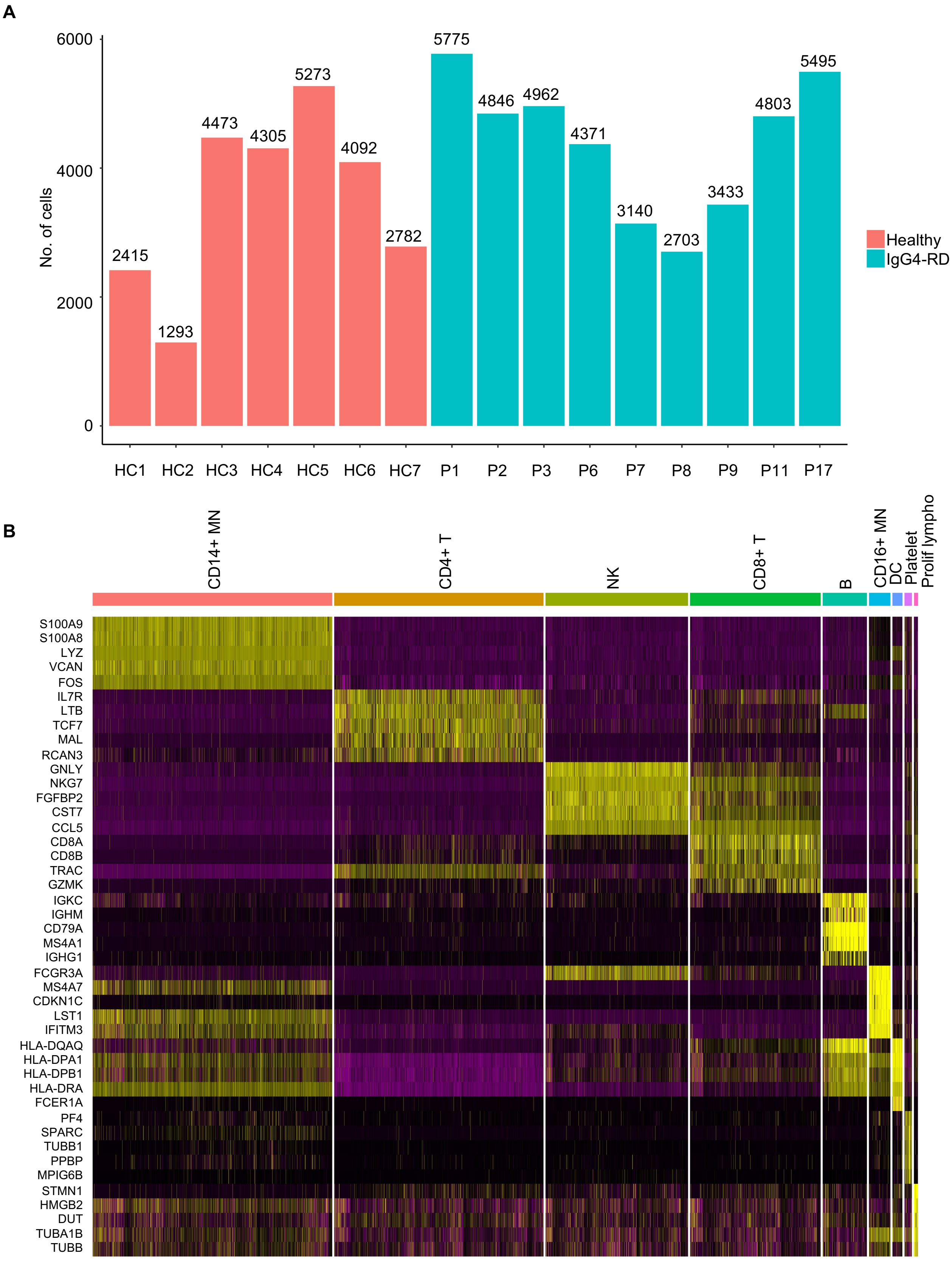

Supplementary Figure 1. **A**, An overview of number of cells of each sample; **B**, Heatmap showing the expression of top 5 significantly enriched transcripts in each cluster (cluster of interest versus all other cells).

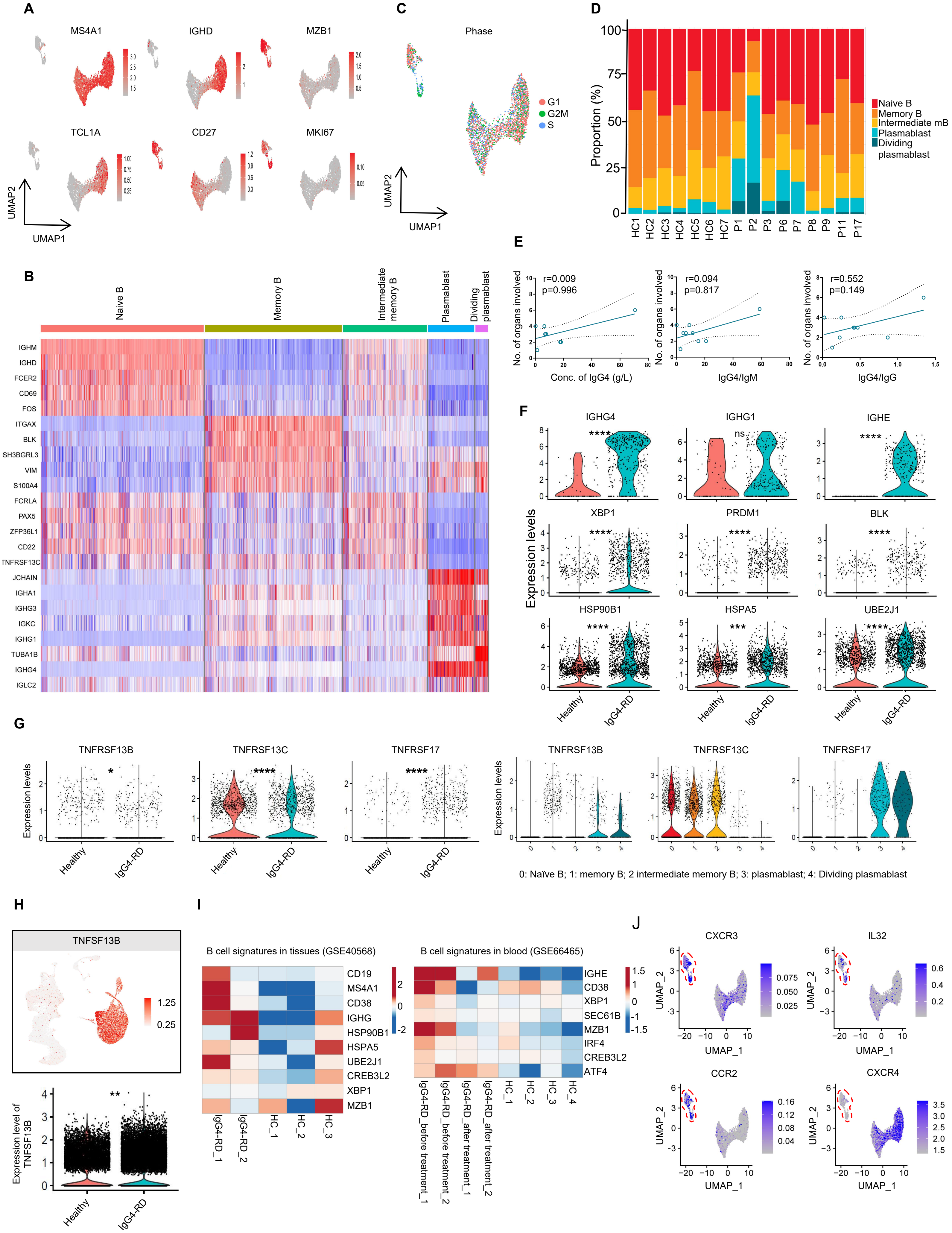
A
MS4A1
IGHD
MZB1
TCL1A
CD27
MKI67
UMAP2
UMAP1
C
Phase
G1
G2M
S
UMAP2
UMAP1
D
Proportion (%)
Naive B
Memory B
Intermediate mB
Plasmablast
Dividing plasmablast
HC1 HC2 HC3 HC4 HC5 HC6 HC7 P1 P2 P3 P6 P7 P8 P9 P11 P17
B
Naive B
Memory B
Intermediate memory B
Plasmablast
Dividing plasmablast
IGHM
IGHD
FCER2
CD69
FOS
ITGAX
BLK
SH3BGRL3
VIM
S100A4
FCRLA
PAX5
ZFP36L1
CD22
TNFRSF13C
JCHAIN
IGHA1
IGHG3
IGKC
IGHG1
TUBA1B
IGHG4
IGLC2
E
r=0.009
p=0.996
r=0.094
p=0.817
r=0.552
p=0.149
No. of organs involved
Conc. of IgG4 (g/L)
IgG4/IgM
IgG4/IgG
F
IGHG4
IGHG1
IGHE
XBP1
PRDM1
BLK
HSP90B1
HSPA5
UBE2J1
Expression levels
Healthy
IgG4-RD
G
TNFRSF13B
TNFRSF13C
TNFRSF17
Expression levels
Healthy
IgG4-RD
0: Naïve B; 1: memory B; 2 intermediate memory B; 3: plasmablast; 4: Dividing plasmablast
H
TNFSF13B
Expression level of TNFSF13B
Healthy
IgG4-RD
I
B cell signatures in tissues (GSE40568)
CD19
MS4A1
CD38
IGHG
HSP90B1
HSPA5
UBE2J1
CREB3L2
XBP1
MZB1
IgG4-RD_1
IgG4-RD_2
HC_1
HC_2
HC_3
B cell signatures in blood (GSE66465)
IGHE
CD38
XBP1
SEC61B
MZB1
IRF4
CREB3L2
ATF4
IgG4-RD_before treatment_1
IgG4-RD_before treatment_2
IgG4-RD_after treatment_1
IgG4-RD_after treatment_2
HC_1
HC_2
HC_3
HC_4
J
CXCR3
IL32
CCR2
CXCR4
UMAP_2
UMAP_1

Supplementary Figure 2. **A**, Expression of selective canonical genes for five major subtypes in B cells. **B,** Heatmap showing the expression of top 5 significantly enriched transcripts in each cluster. **C**, Distribution of cells in different cell phase. **D,** Barplot showing proportions of B subpopulations in each sample. HC: health controls; P: patients. **E**, Scatter plots depicting the correlation between serum concentration of IgG4 (g/L, Spearman's r), IgG4/IgM ratio (Spearman's r), or IgG4/IgG ratio (Pearson's r) and numbers of involved organs in IgG4-RD patients (n=9). Exact two-tailed P values and Pearson's/Spearman's r were presented. **F,** Violin plots showing expression level of immunoglobin-coding genes, B cell activation genes, and ER stress-associated genes in B cells of IgG4-RD and HC. **G,** Violin plots showing expression levels of genes coding receptors for BAFF in B cells of IgG4-RD and HC and in B clusters. **H**, TNFSF13B (encoding BAFF) was specifically expressed in monocytes, and was upregulated in IgG4-RD. **I**, Heatmap showing the expression of B cell maturation-associated genes in tissues (GSE40568) and blood (GSE66465). **J**, Plasmablasts and dividing plasmablasts have upregulated expression of CX3CR1, CCR2, and IL32, but absence of CXCR4 expression. Significance was determined by student's T test or Mann–Whitney test. *p<0.05, **p<0.01, ***p<0.001, ****p<0.0001.

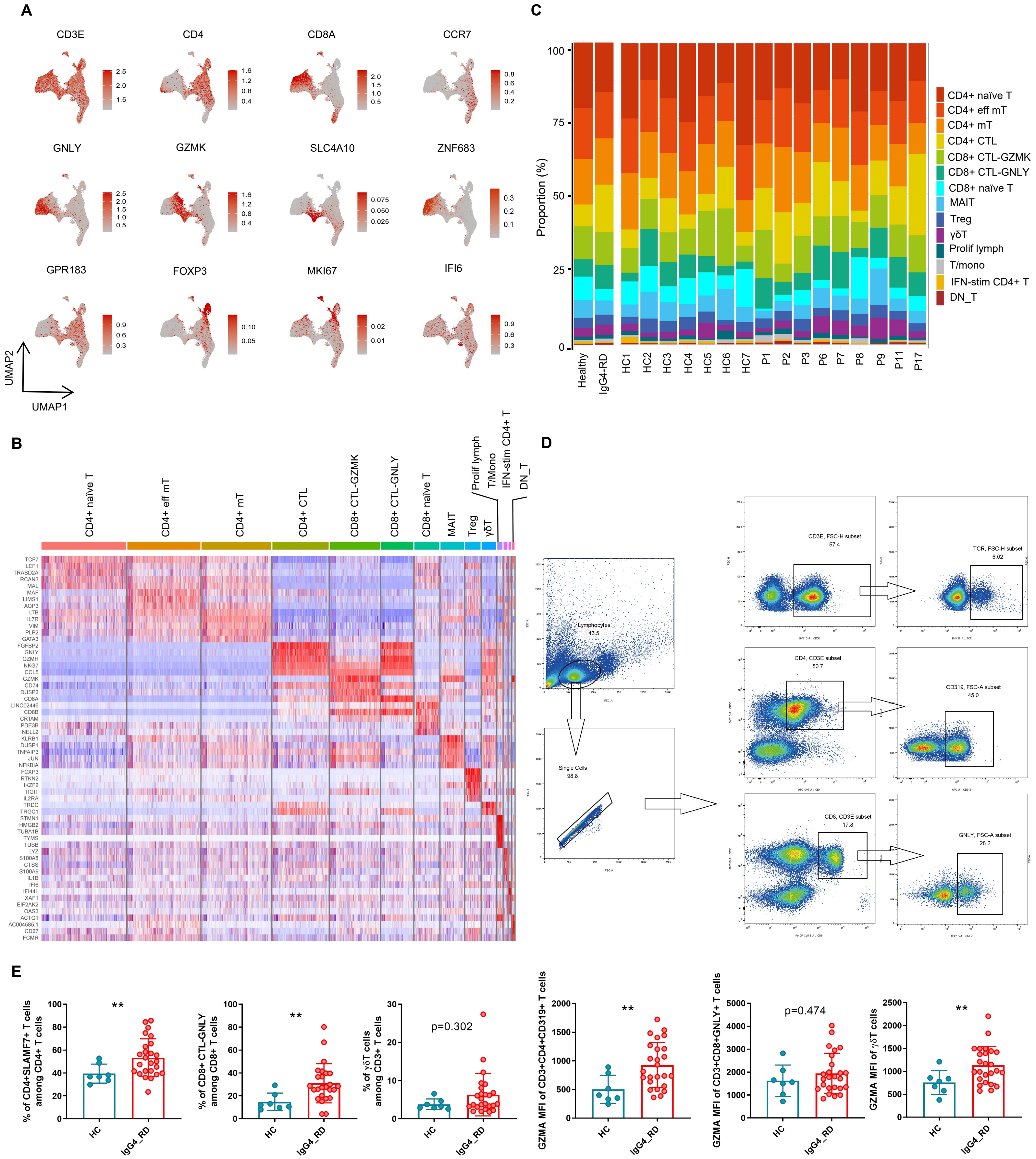
A
CD3E
CD4
CD8A
CCR7
GNLY
GZMK
SLC4A10
ZNF683
GPR183
FOXP3
MKI67
IFI6
UMAP2
UMAP1
C
Proportion (%)
Healthy
IgG4-RD
HC1
HC2
HC3
HC4
HC5
HC6
HC7
P1
P2
P3
P6
P7
P8
P9
P11
P17
CD4+ naïve T
CD4+ eff mT
CD4+ mT
CD4+ CTL
CD8+ CTL-GZMK
CD8+ CTL-GNLY
CD8+ naïve T
MAIT
Treg
γδT
Prolif lymph
T/mono
IFN-stim CD4+ T
DN_T
B
CD4+ naïve T
CD4+ eff mT
CD4+ mT
CD4+ CTL
CD8+ CTL-GZMK
CD8+ CTL-GNLY
CD8+ naïve T
MAIT
Treg
γδT
Prolif lymph
T/Mono
IFN-stim CD4+ T
DN_T
TCF7
LEF1
TRABD2A
RCAN3
MAL
MAF
LIMS1
AQP3
LTB
IL7R
VIM
PLP2
GATA3
FGFBP2
GNLY
GZMH
NKG7
CCL5
GZMK
CD74
DUSP2
CD8A
LINC02446
CD8B
CRTAM
PDE3B
NELL2
KLRB1
DUSP1
TNFAIP3
JUN
NFKBIA
FOXP3
RTKN2
IKZF2
TIGIT
IL2RA
TRDC
TRGC1
STMN1
HMGB2
TUBA1B
TYMS
TUBB
LYZ
CTSS
S100A8
S100A9
IL1B
IFI6
IFI44L
XAF1
EIF2AK2
OAS3
ACTG1
AC004585.1
CD27
FCMR
D
Lymphocytes
43.5
Single Cells
98.8
CD3E, FSC-H subset
67.4
TCR, FSC-H subset
6.02
CD4, CD3E subset
50.7
CD319, FSC-A subset
45.0
CD8, CD3E subset
17.8
GNLY, FSC-A subset
28.2
E
% of CD4+SLAMF7+ T cells among CD4+ T cells
**
% of CD8+ CTL-GNLY among CD8+ T cells
**
% of γδT cells among CD3+ T cells
p=0.302
GZMA MFI of CD3+CD4+CD319+ T cells
**
GZMA MFI of CD3+CD8+GNLY+ T cells
p=0.474
GZMA MFI of γδT cells
**
HC
IgG4_RD

Supplementary Figure 3. **A**, UMAP plots showing the expression of selected canonical genes. **B**, Heatmap showing the expression of top 5 significantly enriched transcripts in each cluster. **C**, Barplot showing proportions of T subpopulations in each sample. HC: health control; P: patient. **D**, Gating strategy for flow cytometry data of lymphocytes. **E**, Proportions and mean florescence intensity of GZMA of each cell type in HCs (n=7) and IgG4-RD patients (n=25) by flow cytometry. Results were shown as mean±SD. Significance was determined by student's T test or Mann–Whitney test. *p<0.05, **p<0.01.

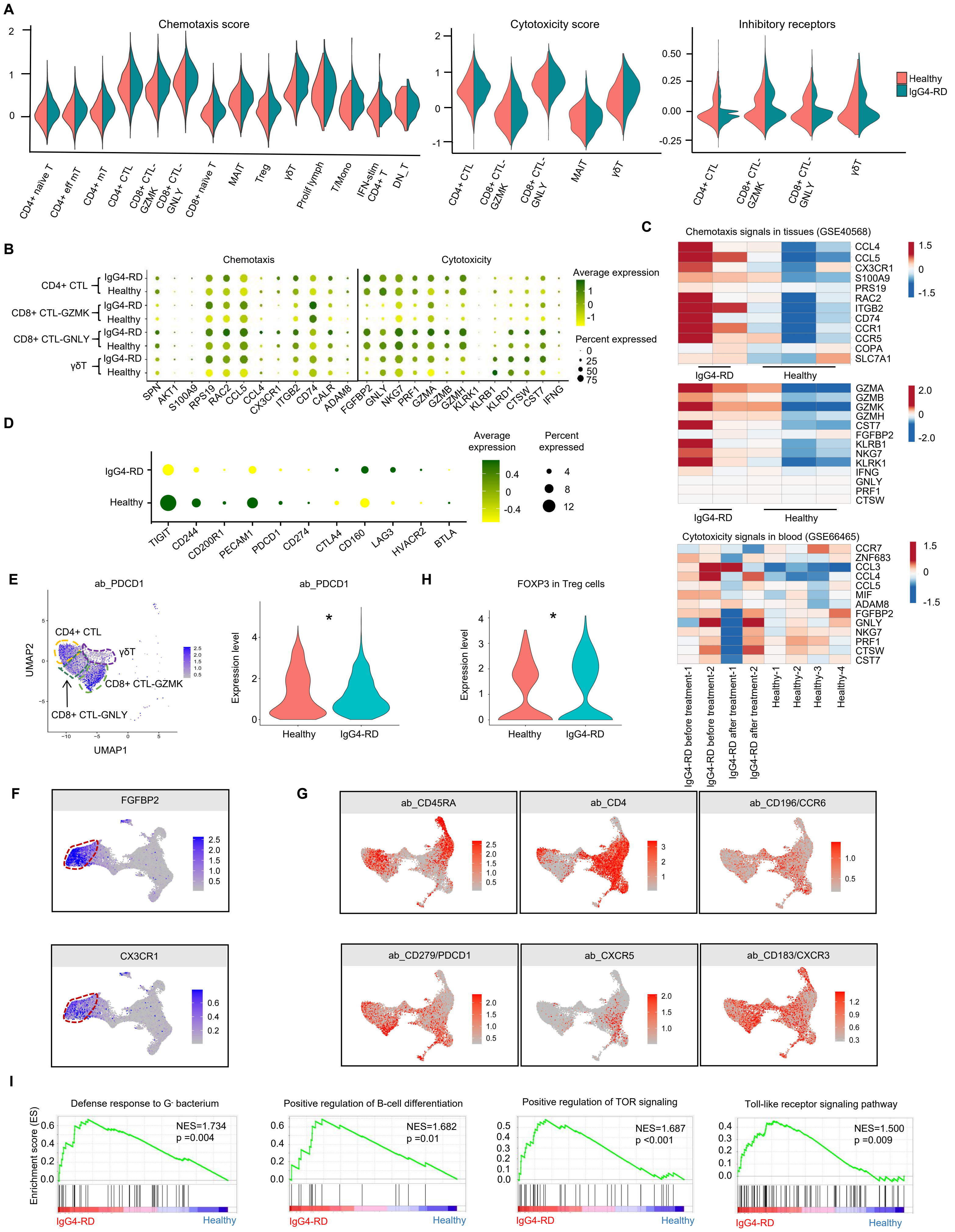
A
Chemotaxis score
Cytotoxicity score
Inhibitory receptors
Healthy
IgG4-RD
CD4+ naive T
CD4+ eff mT
CD4+ mT
CD4+ CTL
CD8+ CTL-GZMK
CD8+ CTL-GNLY
CD8+ naive T
MAIT
Treg
γδT
Prolif lymph
T/Mono
IFN-stim CD4+ T
DN_T
B
Chemotaxis
Cytotoxicity
Average expression
Percent expressed
C
Chemotaxis signals in tissues (GSE40568)
Cytotoxicity signals in blood (GSE66465)
IgG4-RD
Healthy
IgG4-RD before treatment-1
IgG4-RD before treatment-2
IgG4-RD after treatment-1
IgG4-RD after treatment-2
Healthy-1
Healthy-2
Healthy-3
Healthy-4
D
E
ab_PDCD1
UMAP1
UMAP2
Expression level
H
FOXP3 in Treg cells
F
FGFBP2
CX3CR1
G
ab_CD45RA
ab_CD4
ab_CD196/CCR6
ab_CD279/PDCD1
ab_CXCR5
ab_CD183/CXCR3
I
Defense response to G- bacterium
NES=1.734
p =0.004
Positive regulation of B-cell differentiation
NES=1.682
p =0.01
Positive regulation of TOR signaling
NES=1.687
p <0.001
Toll-like receptor signaling pathway
NES=1.500
p =0.009
Enrichment score (ES)

Supplementary Figure 4. **A**, Violin plots showing expression of chemotaxis-associated genes, cytotoxicity-associated genes, and inhibitory receptors shown in Figure 4D. **B**, Dot plot of chemotaxis and cytotoxicity-associated genes in CD4+ CTLs, CD8+ CTLs-GZMK, CD8+ CTLs-GNLY, and γδT cells from IgG4-RD and HCs. **C**, Heatmap showing the expression of chemotaxis and cytotoxicity signals in tissues (GSE40568) and blood (GSE66465) of IgG4-RD and HC. **D**, the expression levels of each inhibitory receptors. **E**, the expression level of PD-1 protein in $CD4^+$ CTLs, $CD8^+$ CTLs and γδT cells and comparison between IgG4-RD and HC. **F**, UMAPs depicts the cytotoxic T cells have high expression of FGFBP2 and CX3CR1. **G**, UMAPs showing the expression of CD45RA, CD4, CD279/PDCD1, CXCR5, CCR6, and CXCR3 protein on T cells. **H**, comparison of Foxp3 expression in Treg cells between IgG4-RD and HCs. **I**, GSEA shows top enriched pathways in T cells. NES denotes normalized enrichment score. Significance was determined by student's T test or Mann–Whitney test. *$p<0.05$.

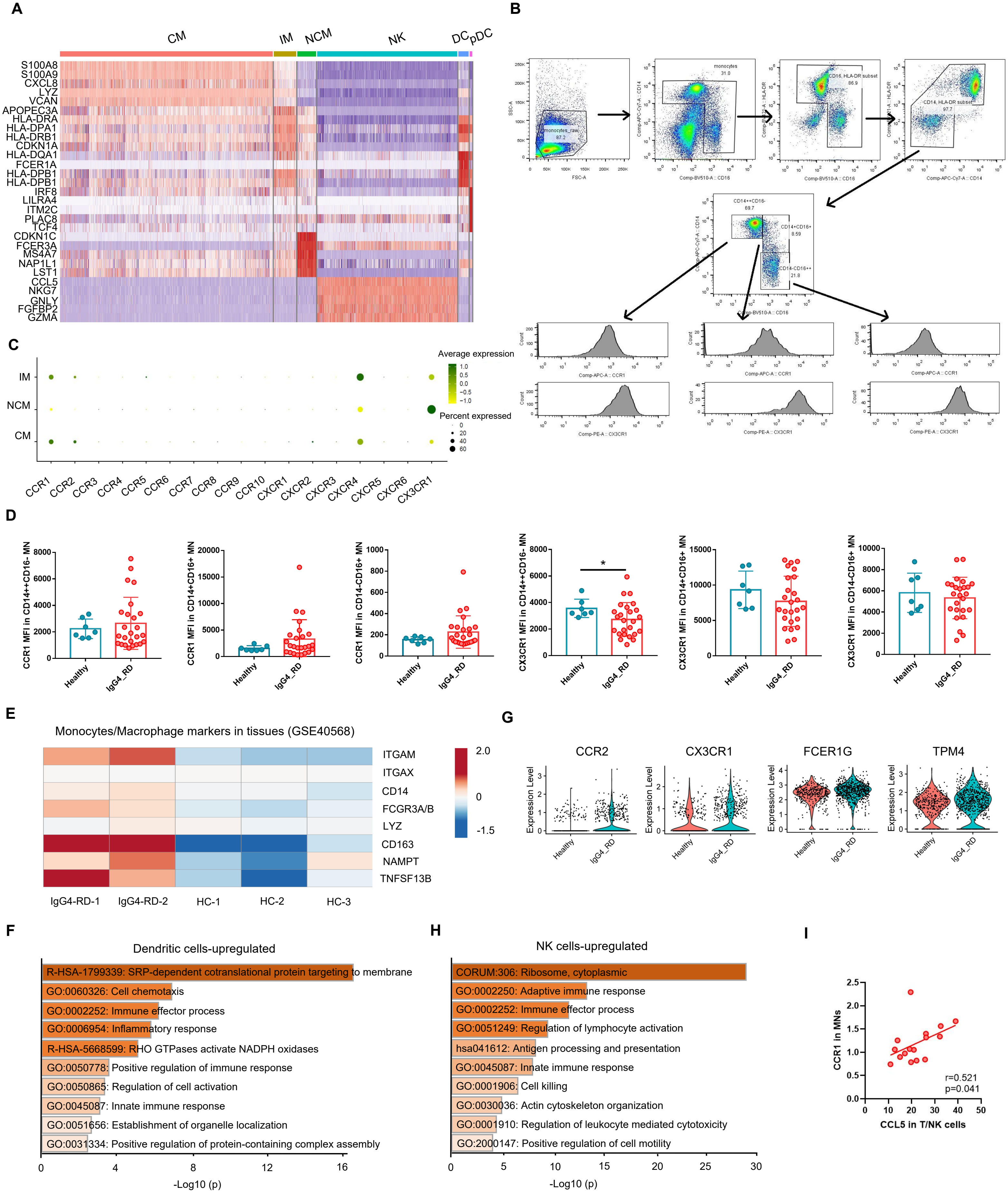
A
CM
IM
NCM
NK
DC
pDC
S100A8
S100A9
CXCL8
LYZ
VCAN
APOPEC3A
HLA-DRA
HLA-DPA1
HLA-DRB1
CDKN1A
HLA-DQA1
FCER1A
HLA-DPB1
HLA-DPB1
IRF8
LILRA4
ITM2C
PLAC8
TCF4
CDKN1C
FCER3A
MS4A7
NAP1L1
LST1
CCL5
NKG7
GNLY
FGFBP2
GZMA
B
monocytes_raw
87.2
SSC-A
FSC-A
monocytes
31.0
Comp-APC-Cy7-A :: CD14
Comp-BV510-A :: CD16
CD16, HLA-DR subset
86.9
Comp-APC-Cy7-A :: HLA-DR
CD14, HLA-DR subset
97.7
CD14++CD16-
69.7
CD14+CD16+
8.59
CD14-CD16++
21.8
Count
Comp-APC-A :: CCR1
Comp-PE-A :: CX3CR1
C
Average expression
1.0
0.5
0.0
−0.5
−1.0
Percent expressed
0
20
40
60
IM
NCM
CM
CCR1
CCR2
CCR3
CCR4
CCR5
CCR6
CCR7
CCR8
CCR9
CCR10
CXCR1
CXCR2
CXCR3
CXCR4
CXCR5
CXCR6
CX3CR1
D
CCR1 MFI in CD14++CD16- MN
CCR1 MFI in CD14+CD16+ MN
CCR1 MFI in CD14-CD16+ MN
CX3CR1 MFI in CD14++CD16- MN
CX3CR1 MFI in CD14+CD16+ MN
CX3CR1 MFI in CD14-CD16+ MN
Healthy
IgG4_RD
*
E
Monocytes/Macrophage markers in tissues (GSE40568)
ITGAM
ITGAX
CD14
FCGR3A/B
LYZ
CD163
NAMPT
TNFSF13B
IgG4-RD-1
IgG4-RD-2
HC-1
HC-2
HC-3
2.0
0
-1.5
G
CCR2
CX3CR1
FCER1G
TPM4
Expression Level
Healthy
IgG4_RD
F
Dendritic cells-upregulated
R-HSA-1799339: SRP-dependent cotranslational protein targeting to membrane
GO:0060326: Cell chemotaxis
GO:0002252: Immune effector process
GO:0006954: Inflammatory response
R-HSA-5668599: RHO GTPases activate NADPH oxidases
GO:0050778: Positive regulation of immune response
GO:0050865: Regulation of cell activation
GO:0045087: Innate immune response
GO:0051656: Establishment of organelle localization
GO:0031334: Positive regulation of protein-containing complex assembly
-Log10 (p)
H
NK cells-upregulated
CORUM:306: Ribosome, cytoplasmic
GO:0002250: Adaptive immune response
GO:0002252: Immune effector process
GO:0051249: Regulation of lymphocyte activation
hsa041612: Antigen processing and presentation
GO:0045087: Innate immune response
GO:0001906: Cell killing
GO:0030036: Actin cytoskeleton organization
GO:0001910: Regulation of leukocyte mediated cytotoxicity
GO:2000147: Positive regulation of cell motility
-Log10 (p)
I
CCR1 in MNs
CCL5 in T/NK cells
r=0.521
p=0.041

Supplementary Figure 5. A, Heatmap showing the expression of top 5 significantly enriched transcripts in each cluster. **B**, Gating strategy for flow cytometry data of monocytes. **C**, Dot plot of chemokine receptor genes in monocytes. **D**, Flow cytometry showing expression of CCR1 and CX3CR1 on CM, IM, and NCM from HCs (n=7) or IgG4-RD patients (n=25). **E**, Heatmap illustrating the expression of MN/macrophage maker genes in IgG4-RD and HC tissues (GSE40568). **F**, Biological processes enriched for upregulated genes in DCs from IgG4-RD versus HC. **G**, Violin plots showing the comparisons of CCR2, CX3CR1, FER1G, and TPM4 genes in IgG4-RD and HC DCs. **H**, Biological processes enriched for upregulated genes in NK cells from IgG4-RD versus HC. **I**, The relationship between CCR1 expression in MNs and CCL5 expression in T and NK cells (n=16, Pearson's r with 2-tailed p value). CM: classical monocyte, NCM: non-classical monocyte, IM: intermediate monocyte, DC: dendritic cell, pDC: plasmacytoid dendritic cell. Results were shown as mean±SD. Significance was determined by student's T test or Mann–Whitney test. *$p<0.05$.

Supplementary Table 1 Characteristics of patients with IgG4-RD and healthy controls

No.	sex	age(year)	Allergy	Lymph node	salivary/lachrymal glands	respiratory system	Liver, biliary tract and pancreas	Treatment at the time of enrollment
P-1	F	69	NA	Lymph nodes in the mediastinum, bilateral axillary, retroperitoneal area were enlarged	None	Patient had recurrent cough. Pulmonary CT scan found fibrosis in some areas of both lungs.	CT showed: 1) the pancreas is round and blunt and enveloped by "capsule" like structure. 2) the liver was slightly larger; the wall of intrahepatic and extrahepatic bile ducts and gallbladder was slightly thicker, and the intrahepatic and extrahepatic bile ducts were slightly dilated; 3) the left anterior and lateral pyramidal renal fascia were thickened.	Amlodipine 5mg daily for hypertension; Gliclazide 60mg daily for diabetes
P-2	M	49	none	Lymph nodes in the cervical, bilateral axillary, mediastinum area were enlarged	Bilateral lachrymal glands, parotid glands, and submandibular glands were swollen	The wall of nasopharynx swelled and bilateral pharyngeal recess disappeared. Pulmonary CT	CT scan found several low-density foci in the left lobe of the liver, thickening of extrahepatic bile duct wall and mild dilatation of intrahepatic bile duct. The body and tail of pancreas were slightly dilated and the spleen was enlarged.	NO

						scan found patchy and nodular shadows in both lungs, and two nodules in the upper lobe of the right lung.		
P-3	F	47	amoxillin, metronidazole, and iodine	Cervical lymph nodes were enlarged	Bilateral lachrymal glands, submandibular, and sublingual glands were enlarged	None	None	NO
P-6	M	69	urticaria history	Cervical lymph nodes were enlarged	Bilateral lachrymal glands, parotid glands, sublingual and submandibular glands were swollen	None	None	NO

P-7	M	74	none	The mediastinum and right axillary lymph nodes were enlarged	Bilateral lachrymal glands were enlarged	Mild obstructive ventilation dysfunction with mild impaired lung function.	None	Acetylcysteine 600mg twice daily and Methoxyphenamine 25mg 3 times/day for cough
P-8	F	37	none	Bilateral inguinal lymph nodes were enlarged	Right lachrymal glands and bilateral submandibular glands were enlarged	New organisms were found in the right nasal cavity.	None	NO
P-9	M	39	none	Bilateral cervical and retroauricular lymph nodes and those near bilateral parotid glands were enlarged	None	Scattered patch and ground glass shadow in both lungs. PET-CT showed abnormal FDG uptake in both lungs.	None	NO

P-11	M	55	bronchial asthma	None	Bilateral lachrymal glands were enlarged	None	None	Amlodipine 5mg daily and Irbesartan 150mg daily for hypertension; Metformin 500mg daily and Acarbose 100mg 3 times/day for diabetes
P-17	M	46	none	Left submandibular lymph nodes were enlarged	Sublingual glands and bilateral lachrymal glands were enlarged	Ground glass shadow on pulmonary CT	None	NO
HC1	F	47	NA	NA	NA	NA	NA	NO
HC2	M	54	NA	NA	NA	NA	NA	NO
HC3	M	49	NA	NA	NA	NA	NA	NO
HC4	F	59	NA	NA	NA	NA	NA	NO
HC5	M	50	NA	NA	NA	NA	NA	NO
HC6	F	45	NA	NA	NA	NA	NA	NO
HC7	M	41	NA	NA	NA	NA	NA	NO

Supplementary Table 2 Laboratory examinations of patients with IgG4-RD

No.	IgG4(g/L)	IgG(g/L)	IgA(mg/L)	IgM(mg/L)	IgE(iu/ml)	kap chain (g/L)	lam chain (g/L)	C3 (g/L)	C4 (g/L)	CRP (mg/L)
P-1	6.37	25.4	6370	702	NA	23.8	9.77	0.437	0.0257	24.4
P-2	70.7	52.6	1200	1200	393	36.7	22.6	0.648	0.139	11.1
P-3	7.7	16.5	2040	1160	158	13.2	7.26	0.875	0.261	3.72
P-6	7.79	18.8	1520	683	893	15.1	8.54	0.462	0.029	1.2
P-7	18.2	21	980	871	NA	15.5	10.2	0.738	0.17	NA
P-8	0.246	10.4	1870	1900	NA	NA	NA	0.759	0.202	NA
P-9	17.6	76.9	6550	1140	1030	53.9	27.7	1.01	0.18	18.9
P-11	1.42	11	2050	513	318	8.67	4.46	0.953	0.278	1.68
P-17	6.93	16.4	2130	1450	<5	12.3	8.69	0.787	0.176	1.6

Red: higher than the normal range; blue: lower than the normal range

Supplementary Table 3 Antibodies conjugated with polyadenylated and antibody-specific barcodes

CD14:MPHIP9-CD14-AHS0037-pAbO	CD45RA:HI100-PTPRC-AHS0009-pAbO
CD16:3G8-FCGR3A-AHS0053-pAbO	CXCR5-CXCR5-AHS0039-pAbO
CD4:SK3-CD4-AHS0032-pAbO	CD279:EH12-1-PDCD1-AHS0014-pAbO
CD8:RPA-T8-CD8A-AHS0027-pAbO	CD196-CCR6-AHS0034-pAbO
CD56:NCAM16.2-NCAM1-AHS0019-pAbO	

References

1. Erickson JR, Mair F, Bugos G, et al. AbSeq Protocol Using the Nano-Well Cartridge-Based Rhapsody Platform to Generate Protein and Transcript Expression Data on the Single-Cell Level. STAR Protoc 2020;1:100092.
2. Qiu X, Mao Q, Tang Y, et al. Reversed graph embedding resolves complex single-cell trajectories. Nat Methods 2017;14:979-82.
3. Aibar S, González-Blas CB, Moerman T, et al. SCENIC: single-cell regulatory network inference and clustering. Nat Methods 2017;14:1083-6.
4. Efremova M, Vento-Tormo M, Teichmann SA, Vento-Tormo R. CellPhoneDB: inferring cell-cell communication from combined expression of multi-subunit ligand-receptor complexes. Nat Protoc 2020;15:1484-506.
5. Jin S, Guerrero-Juarez CF, Zhang L, et al. Inference and analysis of cell-cell communication using CellChat. Nat Commun 2021;12:1088.
